# Supplementary material for: Timescale correlation of shallow trap states increases electrochemiluminescence efficiency in carbon nitrides
Source: Nat Commun. 2024 Apr 27;15:3597. doi: 10.1038/s41467-024-48011-y (PMC11519465; doi:10.1038/s41467-024-48011-y)
Supplement: Supplementary file 1 — Supplementary Information [file 41467_2024_48011_MOESM1_ESM.pdf]

Supplementary Information

Timescale Correlation of Shallow Trap States Increases Electrochemiluminescence Efficiency in Carbon Nitrides

Yanfeng Fang[1,4], Hong Yang[1,4], Yuhua Hou[1], Wang Li[1], Yanfei Shen[2*], Songqin Liu[1], Yuanjian Zhang[1,3]*

[1] Jiangsu Engineering Research Center for Carbon-Rich Materials and Devices, Jiangsu Province Hi-Tech Key Laboratory for Bio-Medical Research, School of Chemistry and Chemical Engineering, Nanjing 211189, China

[2]Medical School, Southeast University, Nanjing 210009, China.

[3]Department of Oncology, Zhongda Hospital, Southeast University, Nanjing 210009, China.

[4]These authors contributed equally: Yanfeng Fang, Hong Yang.

E-mail: Yanfei.Shen@seu.edu.cn (Y.S.), Yuanjian.Zhang@seu.edu.cn (Y.Z.)

Table of Contents

Name	Page
**Supplementary Fig. 1.** Top (a, and c) and cross-sectional (b and d) SEM images of CN and Au_x-CN photoelectrodes.	5
**Supplementary Fig. 2.** Scratch morphologies of the respective tracks for the CN and Au_x-CN photoelectrodes.	6
**Supplementary Fig. 3.** High-resolution STEM-EDS elemental mapping images of C, N and Au of Au_x-CN.	7
**Supplementary Fig. 4.** FTIR of CN and Au_x-CN photoelectrodes.	8
**Supplementary Fig. 5.** High-resolution C 1*s* (a and c) and N 1*s* (b and d) XPS spectra of CN and Au_x-CN. "a.u." refers to arbitrary units.	9
**Supplementary Fig. 6.** LDI-TOF mass spectra of CN and Au_x-CN.	10
**Supplementary Fig. 7.** (a) VB-XPS, (b) UV-vis, (c) Kubelka-Munk plot and (d) energy level diagram of CN, Au_x-CN and bulk CN_{550}.	11
**Supplementary Fig. 8.** Normalized Au L3-edge XANES spectra Au_x-CN and the reference samples.	12
**Supplementary Fig. 9.** High-resolution Au 4*f* XPS spectra of Au_x-CN and Au_{NPs}/CN photoelectrodes. "a.u." refers to arbitrary units.	13
**Supplementary Fig. 10.** LSV curve simulated of (a) CN photoelectrode with R= 395 ohm and (b) Au_x-CN photoelectrode with R= 195 ohm.	14
**Supplementary Fig. 11.** (a) ECL spectrum and (b) ECL intensity of Au_x-CN photoelectrodes with various Au loading in preparation.	15
**Supplementary Fig. 12.** (a) ECL spectra of Au_x-CN photoelectrode under -1.5 V vs. Ag/AgCl that was used for calculating the total number of photons. (b) Amperometric Q-t curve for ECL reaction of Au_x-CN photoelectrode at -1.5 V vs. Ag/AgCl in 0.01 M phosphate buffer saline, 0.1 M KCl with 25 mM $K_2S_2O_8$ (red line) and without $K_2S_2O_8$ (black line). (c) Number of emitted photons and charges consumed by Faraday reactions.	16
**Supplementary Fig. 13.** (a) ECL spectra of $Ru(bpy)_3Cl_2$ under -1.5 V vs. Ag/AgCl that was used for calculating the total number of photons. (b) Amperometric Q-t curve for ECL reaction of $Ru(bpy)_3Cl_2$ at -1.5 V in 0.01 M phosphate buffer saline, 0.1 M KCl with 25 mM $K_2S_2O_8$. (c) The number of emitted photons and charges consumed by Faraday reactions.	17
**Supplementary Fig. 14.** Schemes for the reaction mechanisms of the $Ru(bpy)_3^{2+}$/TPrA system.	18
**Supplementary Fig. 15.** (a) Nyquist plots for FTO, CN and Au_x-CN photoelectrode in 5 mM $[Fe(CN)_6]^{3-}/[Fe(CN)_6]^{4-}$ containing 0.1 M KCl; (b) the corresponding magnification of FTO in (a).	19
**Supplementary Fig. 16.** Thermal images of CN and Au_x-CN photoelectrodes showing the photothermal effect without (a) or with (b) irradiation time for 10 min.	20
**Supplementary Fig. 17.** Nyquist plots of (a) the CN and (c) Au_x-CN photoelectrodes at different applied potentials versus Ag/AgCl in 0.01 M phosphate buffer saline containing 0.1 M KCl and 25 mM $K_2S_2O_8$. (b) the corresponding magnification of CN in (a). (d) the corresponding magnification of Au_x-CN in (c). Globules were test data points and solid lines were the fitting data.	21

Supplementary Fig. 18. (a) Possible charge transfer processes of ECL in Au_x-CN. (b) Proposed full equivalent circuit used for interpretation of CN and Au_x-CN photoelectrodes. 22

Supplementary Fig. 19. Fitted data for R_t at different overpotentials (-0.9 V~ -1.5 V). 23

Supplementary Fig. 20. OCP decay curves (black) and fitted analysis (red) of (a) CN, (b) Au_x-CN photoelectrodes. 24

Supplementary Fig. 21. Value of (a) k_1, k_2 and (b) ΔV_1, ΔV_2 for CN and Au_x-CN photoelectrodes obtained from OCP. 25

Supplementary Fig. 22. Bode phase plots of (a) CN and (b) Au_x-CN photoelectrodes at different potentials in 0.01 M phosphate buffer saline containing 0.1 M KCl and 25 mM $K_2S_2O_8$. 26

Supplementary Fig. 23. Bias dependence of characteristic lifetime τ_n extracted from mediate frequency EIS spectra of CN and Au_x-CN photoelectrodes. 27

Supplementary Fig. 24. Fitted data for R_{ct} at different overpotentials (-0.2 V~ -1.5 V) of CN and Au_x-CN photoelectrodes. 28

Supplementary Fig. 25. Electron-hole recombination efficiency of CN and Au_x-CN photoelectrodes evaluated by PEC current under chopped light biased at -0.3 V vs. Ag/AgCl in 0.1 M KCl. 29

Supplementary Fig. 26. Visible femtosecond transient absorption spectra of (a) CN and (b) Au_x-CN photoelectrodes at selected time points from 10 ps to 1 ns. 30

Supplementary Fig. 27. Time-resolved FL decay spectra of CN and Au_x-CN photoelectrodes under 365 nm excitation. 31

Supplementary Fig. 28. Possible mechanism for ECL of (a) CN photoelectrode and (b) Au_x-CN photoelectrode with different timescale. 32

Supplementary Fig. 29. ECL emission intensity of different control Au/CN photoelectrodes made by (a) $NaBH_4$ reduction, (c) photoreduction, (e) calcination, and (g) drop cast methods. ECL emission intensity change value (I/I_0) of different control Au/CN photoelectrodes made by (b) $NaBH_4$ reduction, (d) photoreduction, (f) calcination, and (h) drop cast methods. 33

Supplementary Fig. 30. ECL emission intensity change (I/I_0) of Au_x-CN photoelectrode and various control Au/CN photoelectrodes. "a.u." refers to arbitrary units. 34

Supplementary Fig. 31. High-resolution Au 4*f* XPS spectra of Au/CN photoelectrode. 35

Supplementary Fig. 32. Top-sectional SEM images of (a) Au_x-CN and (b) Au/CN photoelectrodes. "a.u." refers to arbitrary units. 36

Supplementary Fig. 33. Structure model of (a) Au_x-CN and (b) various Au/CN. Gray coloring indicates carbon atoms, blue indicates nitrogen, and orange indicates gold. 37

Supplementary Fig. 34. Two possible simplified Au_x-CN models. (a) Au atoms were bonded between two layers of CN, and (b) Au atoms are inserted into the hole in a CN layer. Gray coloring indicates carbon atoms, blue indicates nitrogen, and orange indicates gold. 38

Supplementary Fig. 35. Interlayer charge transfer in Au_x-CN. Charge distribution analysis of (a, b) CN and (c, d) Au_x-CN. $|\Delta q|$ represents the absolute value of the difference in the electron distribution between the layers. The stick model described CN, gray coloring indicates carbon atoms, blue indicates nitrogen, and orange indicates gold. Yellow and blue represent electron accumulation and electron depletion, respectively. 39

Supplementary Fig. 36. ECL intensity of the as-proposed biosensor for detection of NO_2^- under consecutive cyclic potential scanning. "a.u." refers to arbitrary units. 40

Supplementary Fig. 37. Ratio of ECL intensity for Ag_x-CN to CN photoelectrode. 41
Supplementary Table 1. Comparison of the relative co-reactant ECL efficiency of different lumiphores by considering the number of generated photons and consumed electrons (Eq. 7). 42
Supplementary Table 2. Impedance fitting data for FTO, CN and Au_x-CN photoelectrodes in 5 mM $[Fe(CN)_6]^{3-}/[Fe(CN)_6]^{4-}$ and 0.1 M KCl at open circuit potential (-0.23 V). 45
Supplementary Table 3. Summary of the impedance fitting data for CN photoelectrode in 0.01 M phosphate buffer saline containing 0.1 M KCl and 25 mM $K_2S_2O_8$ at different potential. 46
Supplementary Table 4. Summary of the impedance fitting data for Au_x-CN photoelectrode in 0.01 M phosphate buffer saline containing 0.1 M KCl and 25 mM $K_2S_2O_8$ at different potential. 47
Supplementary Table 5. Comparison of the NO_2^- sensing performance. 48
Supplementary references 49

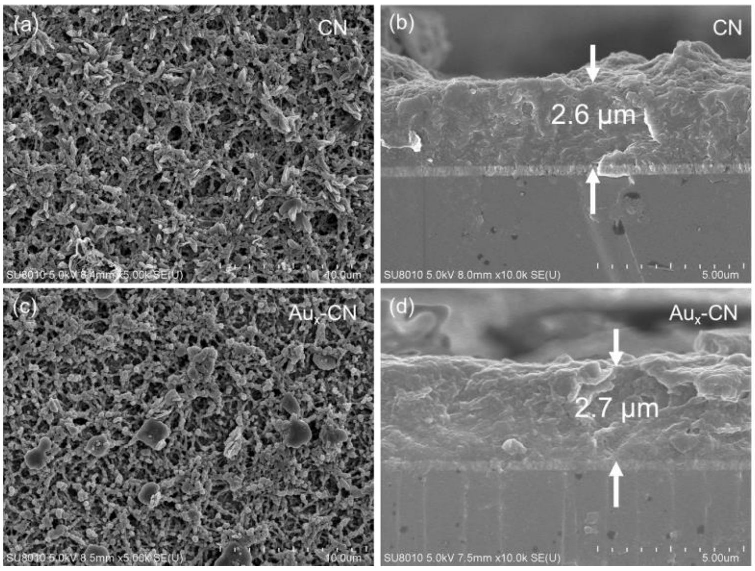

Supplementary Fig. 1 Top (a and c) and cross-sectional (b and d) SEM images of CN and Au_x-CN photoelectrodes.

The high-quality CN and Au_x-CN thin films were obtained by crystallization on FTO and in situ thermal condensation. As shown in Supplementary Fig. 1, a continuous CN and Au_x-CN layer intimately connected to FTO was obtained with a layer thickness of ~3 μm.

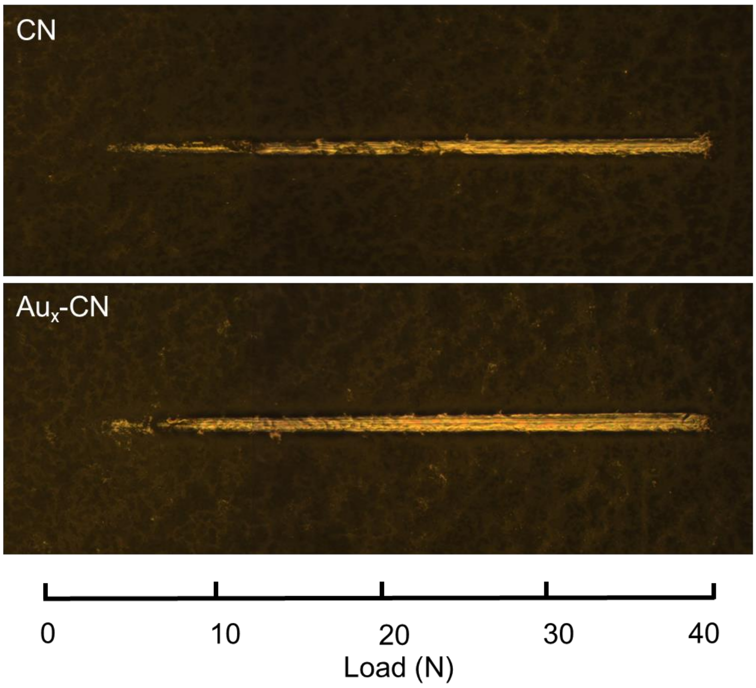

Supplementary Fig. 2 Scratch morphologies of the respective tracks for the CN and Au$_x$-CN photoelectrodes.

To evaluate the toughness of the coatings qualitatively, the scratch morphology of the tracks was measured. As shown in Supplementary Fig. 2, only a few small pieces of debris were observed at the edge of the track, and no obvious chipping events were observed, indicating the good toughness of the CN and Au$_x$-CN photoelectrodes.

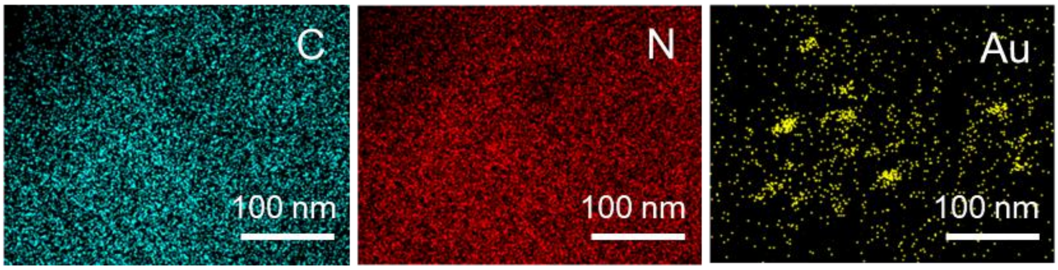

Supplementary Fig. 3 High-resolution STEM-EDS elemental mapping images of C, N and Au of Au_x-CN.

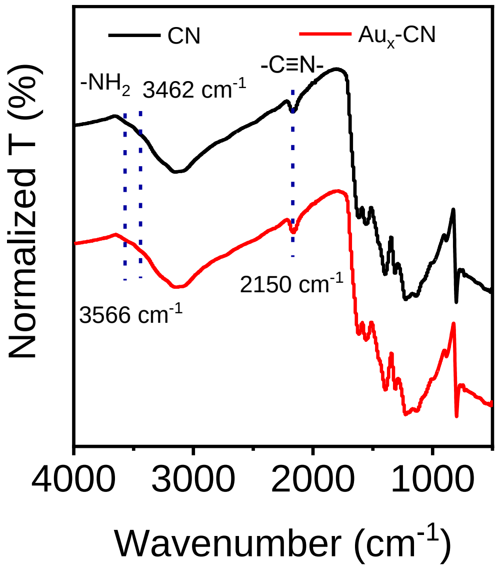

Supplementary Fig. 4 FTIR of CN and Au$_x$-CN photoelectrodes.

As shown in Supplementary Fig. 4, for both CN and Au$_x$-CN photoelectrodes showed the characteristic vibrations peaks for ν(−NH$_2$) at 3566/3462 cm^{-1} and -C≡N- at 2150 cm^{-1}, respectively[1]. Therefore, the type of the terminal groups (such as −NH$_2$ and -C≡H-) on the surface of carbon nitride remained unchanged after introduction of Au species.

In addition, to more accurate comparison of the peak areas of specific functional groups across different samples, the peaks representing the triazine or heptazine ring out of plane bending at 825 cm^{-1} were normalized. The peak area of ν(−NH$_2$) at 3566 cm^{-1}/3462 cm^{-1} for CN and Au$_x$-CN photoelectrodes were 12727/7563 and 12734/6709, the peak area of ν(-C≡N-) at 2150 cm^{-1} for CN and Au$_x$-CN photoelectrodes were 9289 and 9008, respectively. Based on the quantitative data analysis, the quantity of the terminal groups (such as -NH$_2$ and -C≡H-) on the surface of carbon nitride was almost unchanged after the introduction of Au species.

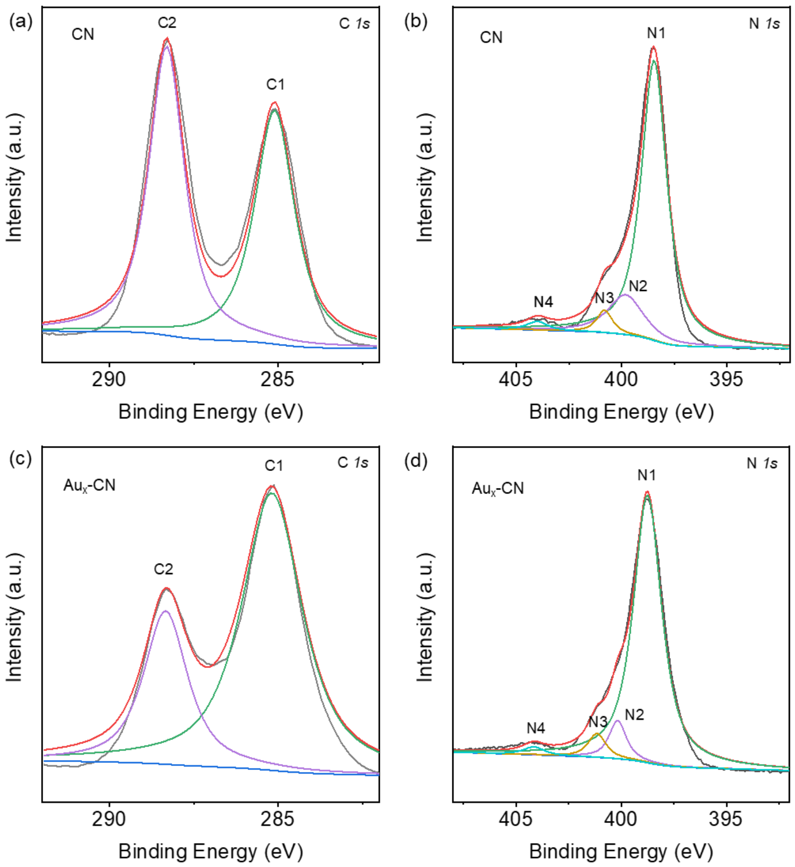

Supplementary Fig. 5 High-resolution C 1*s* (a and c) and N 1*s* (b and d) XPS spectra of and Au_x-CN. "a.u." refers to arbitrary units.

The chemical structure of the CN and Au_x-CN was further confirmed by XPS. The C 1*s* XPS spectra showed the predominant C2 peak at around 288.3 eV for all samples, corresponding to the typical aromatic C−N=C coordination in a CN framework. The C2 peak at 284.6 eV was ascribed to adventitious carbon[2,3]. The high-resolution N 1*s* spectra could be deconvoluted into four peaks with binding energies at around 398.48 (N1, sp^2 bonded nitrogen in N-containing aromatic rings (C−N=C), 399.82 (N2, tertiary nitrogen N-(C)3 groups), 400.79 (N3, amino group (C−N−H)) and 403.93 eV (N4, charging effects or positive charge localization in heterocycles)[2,3].

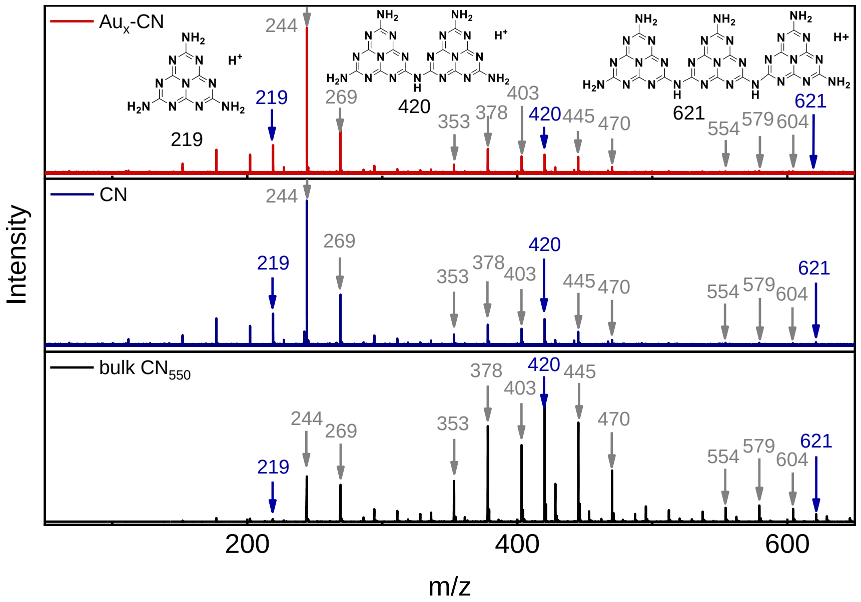

Supplementary Fig. 6 LDI-TOF mass spectra of CN and Au_x-CN.

To disclose more detailed information of molecular structures, especially for the exact type of repeating units, the matrix-free LDI-TOF-MS spectra of CN, Au_x-CN and the control bulk CN_{550} powder were further measured. Interestingly, the LDI-TOF-MS spectra of CN and Au_x-CN photoelectrode showed 219, 420, and 621 m/z fragments, the same as that of bulk CN_{550}, with an interval of 201. It corresponded to the successive loss of protonated heptazine in ionization. A series of peaks with m/z separated by 17, 42, 67, 151, 176, and 201 were also observed, showing the progressive ionization of heptazine to cyanamide[2].

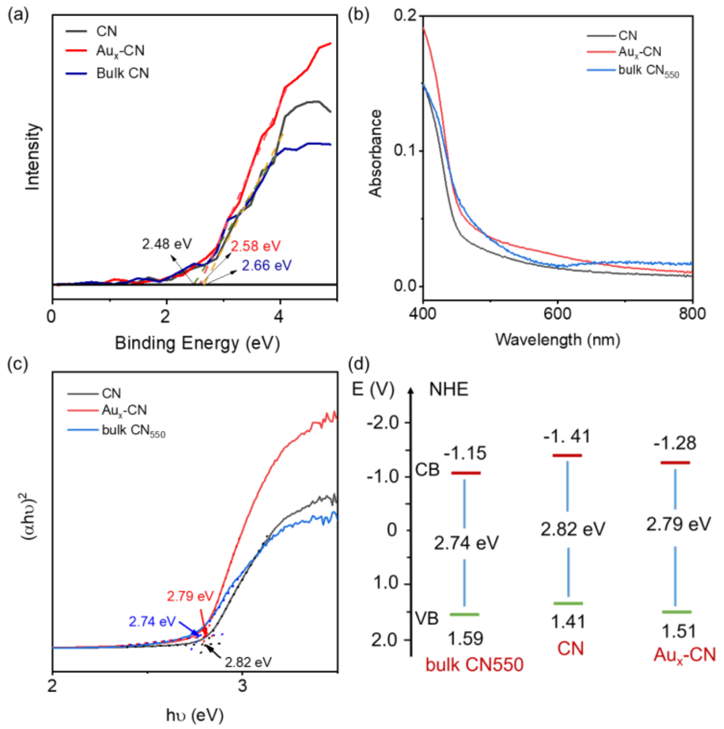

Supplementary Fig. 7 (a) VB-XPS, (b) UV-vis, (c) Kubelka-Munk plot and (d) energy level diagram of CN, Au_x-CN and bulk CN_{550}.

The energy band structure (Supplementary Fig. 7d) of CN and Au_x-CN was further calculated by the relative valence band position obtained from the XPS spectra (Supplementary Fig. 7a) and the band gap obtained from the ultraviolet-visible (UV-vis) spectra (Supplementary Fig. 7b). The changes in the energy band structure of CN and Au_x-CN were due to the incorporation of the Au species into CN.

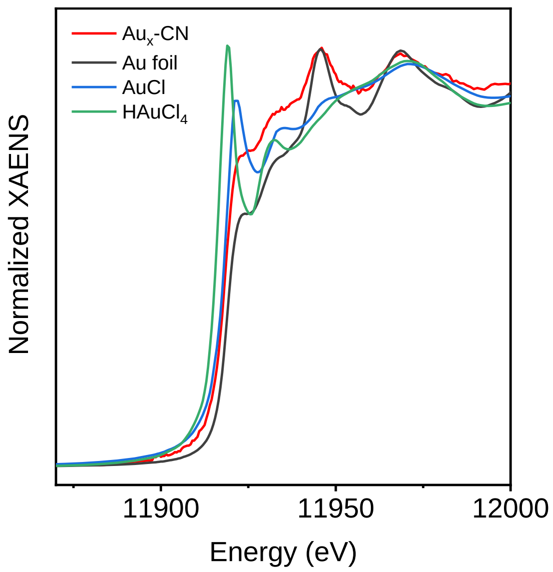

Supplementary Fig. 8 Normalized Au L3-edge XANES spectra of Au_x-CN and the reference samples.

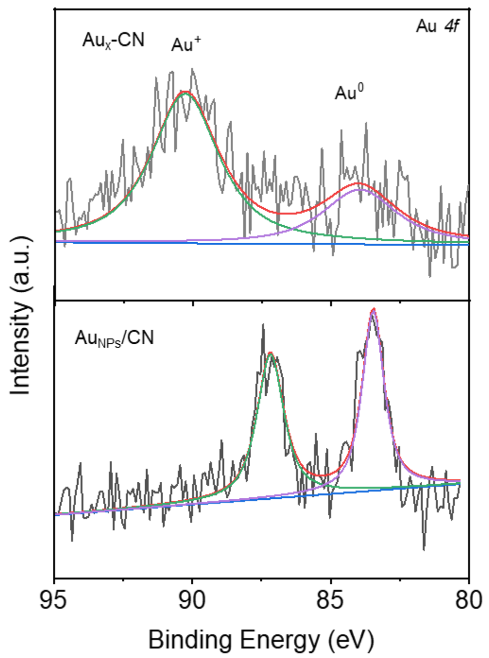

Supplementary Fig. 9 High-resolution Au 4*f* XPS spectra of Au_x-CN and Au_{NPs}/CN photoelectrodes. “a.u.” refers to arbitrary units.

The XPS spectra of Au_x-CN showed the typical $4f^{5/2}$ and $4f^{7/2}$ signals at 90.34 and 83.94 eV, assigning to the oxidation state (Au^I) and metallic state (Au^0), respectively. In contrast, only XPS peaks at a binding energy of 87.21 and 83.49 eV, corresponding to the metallic Au^0 $4f^{5/2}$ and $4f^{7/2}$, were observed for the control Au NPs functionalized CN.

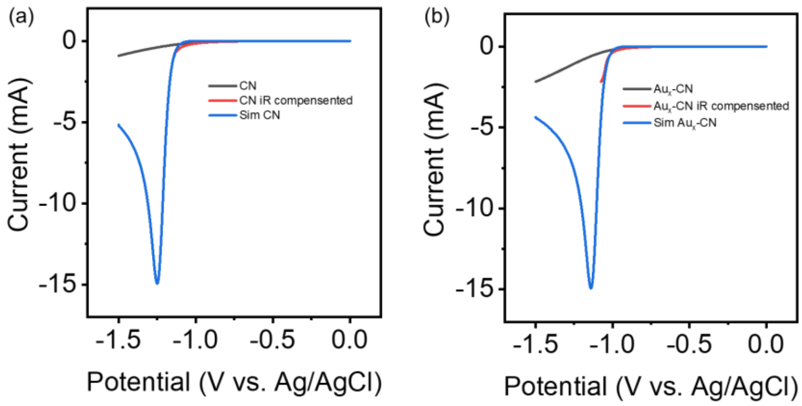

Supplementary Fig. 10 LSV curve simulated of (a) CN photoelectrode with R= 395 ohm and (b) Au_x-CN photoelectrode with R= 195 ohm.

In order to explore the kinetic process of $K_2S_2O_8$ reduction, the LSV curves during the ECL process were simulated by COMSOL Multiphysics. During the simulation process, it was challenging to fit the original LSV curves of CN and Au_x-CN photoelectrode (black curve in Supplementary Fig. 10) with simulation due to the different shapes of the curve around the onset potential. Considering the high resistantcy of the electrode material, the iR compensation was implemented, which assumed a constant resistance R of the electrode. After iR compensation, the LSV curves of CN and Au_x-CN photoelectrode could be fitted with simulation (Supplementary Fig. 10). An important point to note is that only a proper *R* value could lead to a good fit with simulation; too large or too small *R* values would lead to a heavily distorted shape of the LSV (data not shown), which is impossible to fit. Such "ill"-defined CV curves were often ascribed to the nature of electrode materials. First of all, the control CV/ECL curves without $K_2S_2O_8$ (Fig. 2a) demonstrated a minor current, indicating negligible polarization of water during the reduction of $K_2S_2O_8$. Moreover, the finite element analysis (Supplementary Fig. 10) showed the high iR drop made the reduction wave out of the scope of the electrochemical window. In contrast, the reduction wave for $K_2S_2O_8$ could be occasionally observed by low-quality CN film electrodes owing to pinholes, but which had a much lower Φ_{ECL}. It evidently suggested the transferring of the electron to $K_2S_2O_8$ bridged by the CN film could make more use of electrons in ECL, compared to the direct accessing of electrons from the substrate electrode that was independent of the follow-up ECL reactions and generally superfluous.

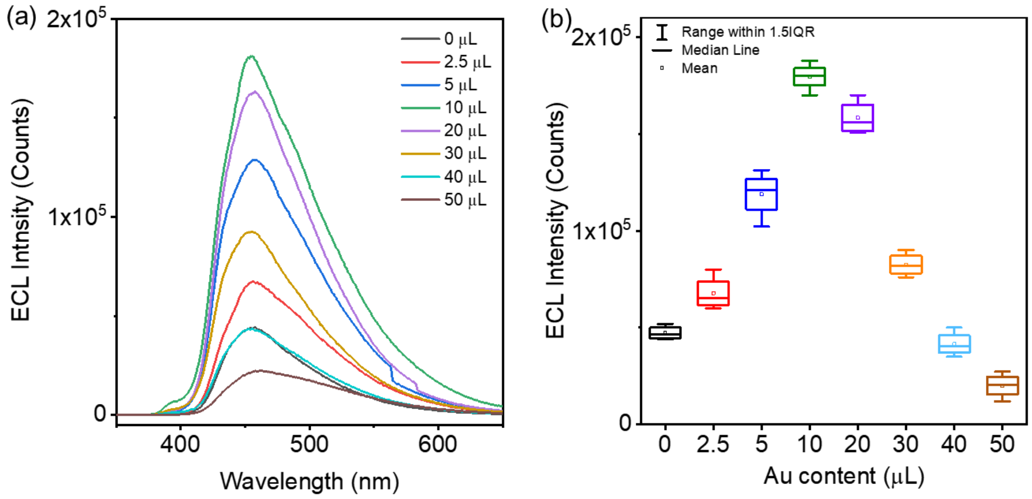

Supplementary Fig. 11. (a) ECL spectrum and (b) ECL intensity of Au_x-CN photoelectrodes (sample number (*n*) is 4 in each group) with various Au loading in preparation. The boxplots display the interquartile range (boxes), the median (horizontal lines in the middle), the 25th and 75th percentile (horizontal lines in the bottom and top) and the mean (square).

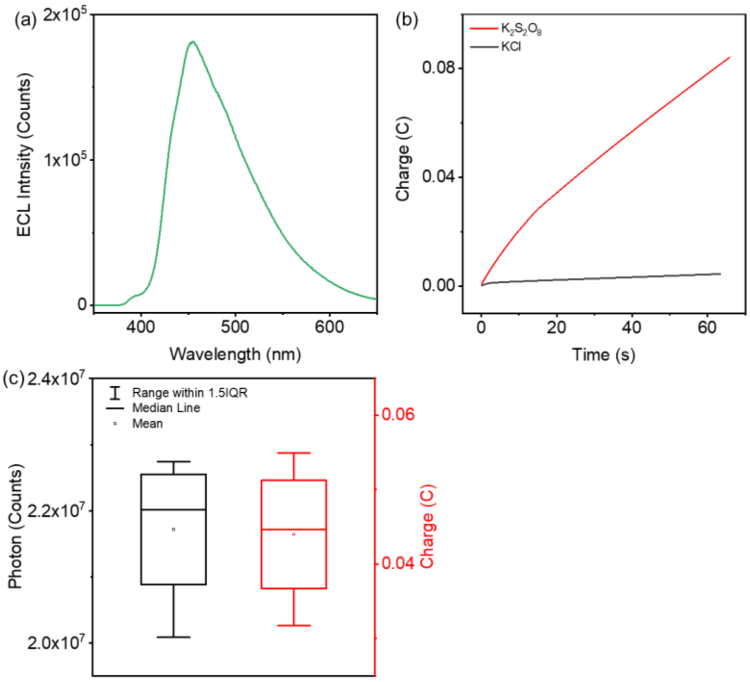

Supplementary Fig. 12 (a) ECL spectra of Au_x-CN photoelectrode under -1.5 V vs. Ag/AgCl that was used for calculating the total number of photons. (b) Amperometric Q-t curve for ECL reaction of Au_x-CN photoelectrode at -1.5 V vs. Ag/AgCl in 0.01 M phosphate buffer saline, 0.1 M KCl with 25 mM $K_2S_2O_8$ (red line) and without $K_2S_2O_8$ (black line). (c) Number of emitted photons and charges consumed by Faraday reactions. The boxplots display the interquartile range (boxes), the median (horizontal lines in the middle), the 25th and 75th percentile (horizontal lines in the bottom and top) and the mean (square), $n = 4$ per group.

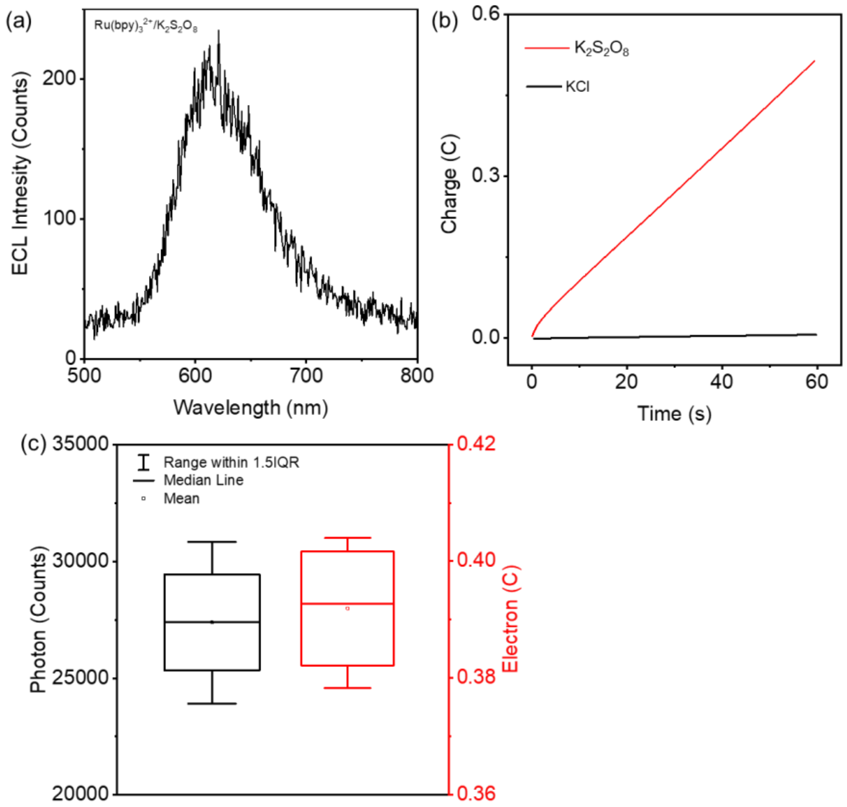

Supplementary Fig. 13 (a) ECL spectra of $Ru(bpy)_3Cl_2$ under -1.5 V vs. Ag/AgCl that was used for calculating the total number of photons. (b) Amperometric Q-t curve for ECL reaction of $Ru(bpy)_3Cl_2$ at -1.5 V in 0.01 M phosphate buffer saline, 0.1 M KCl with 25 mM $K_2S_2O_8$ (red line) and without $K_2S_2O_8$ (black line). (c) The number of emitted photons and charges consumed by Faraday reactions. The boxplots display the interquartile range (boxes), the median (horizontal lines in the middle), the 25th and 75th percentile (horizontal lines in the bottom and top) and the mean (square), $n = 4$ per group.

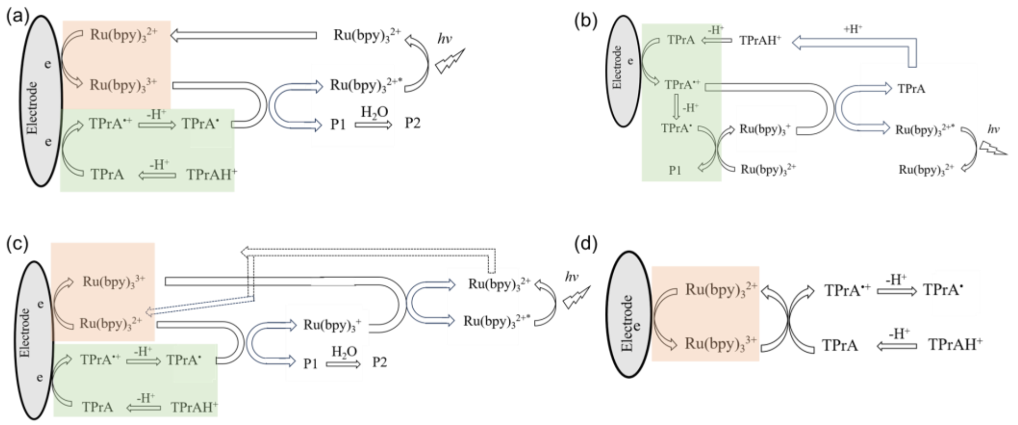

Supplementary Fig. 14 Schemes for the reaction mechanisms of the $Ru(bpy)_3^{2+}$/TPrA system. (a) Electrogenerated $Ru(bpy)_3^{3+}$ reacts with $TPrA^{\bullet}$ from the direct reaction of TPrA at the electrode; (b) Electrogenerated $TPrA^{\bullet+}$ reacts with $Ru(bpy)_3^{+}$; (c) $Ru(bpy)_3^{2+}$ reacts with $TPrA^{\bullet}$ to form $Ru(bpy)_3^{+}$, which can then interact with $Ru(bpy)_3^{3+}$ to form ECL via annihilation; (d) Electrogenerated $Ru(bpy)_3^{3+}$ at the electrode reacts with TPrA. The orange region: emitter directly obtained the electron from the electrode, the green region: co-reagent directly obtained the electron from the electrode.

For co-reactant typed ECL, based on the pathways through which oxidation-reduction reactions occur, they can be categorized into three categories: (1) the co-reactant and the emitters react simultaneously at the electrode (Supplementary Figs. 14a and c); (2) the co-reactants react initially, after which the emitter reacts with the intermediate of the co-reactants (Supplementary Fig. 14b); (3) the emitters react first, followed by an oxidation-reduction reaction between the co-reactant and the emitters (Supplementary Fig. 14d). To elucidate these processes, the most classic ECL system ($Ru(bpy)_3^{2+}$/TPrA) was employed in the literature by Bard and co-workers.

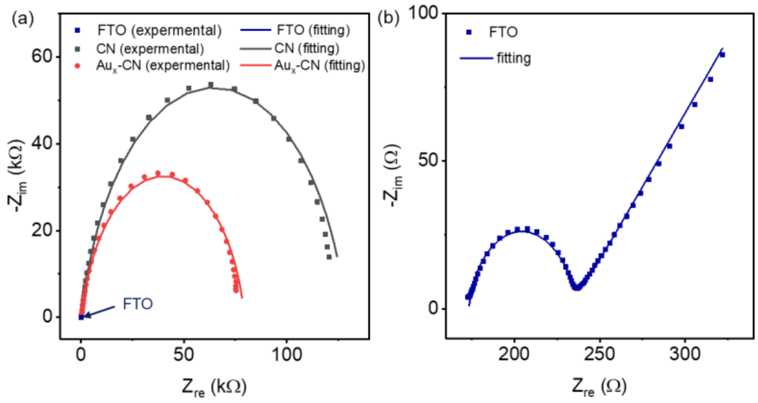

Supplementary Fig. 15 (a) Nyquist plots for FTO, CN and Au_x-CN photoelectrode in 5 mM $[Fe(CN)_6]^{3-}/[Fe(CN)_6]^{4-}$ containing 0.1 M KCl; (b) the corresponding magnification of FTO in (a).

The electrochemical impedance spectra (EIS) of Au_x-CN and FTO photoelectrodes were measured using $[Fe(CN)_6]^{3-}/[Fe(CN)_6]^{4-}$ as the electrochemical probe (Supplementary Fig. 15 and Supplementary Table 2). The redox reaction was evidently inhibited by ca. 4 orders at Au_x-CN photoelectrode in regarding of the interfacial charge transfer resistance across the electrode/electrolyte (R_{ct}), verifying the $Fe(CN)_6]^{3-}$ obtained electron from Au_x-CN instead of the FTO. Therefore, the Au_x-CN/$K_2S_2O_8$ system in this study was followed by the third type of reaction pathway (Supplementary Fig. 15d), i.e., the emitters react first, followed by an oxidation-reduction reaction between the co-reactant and the emitters.

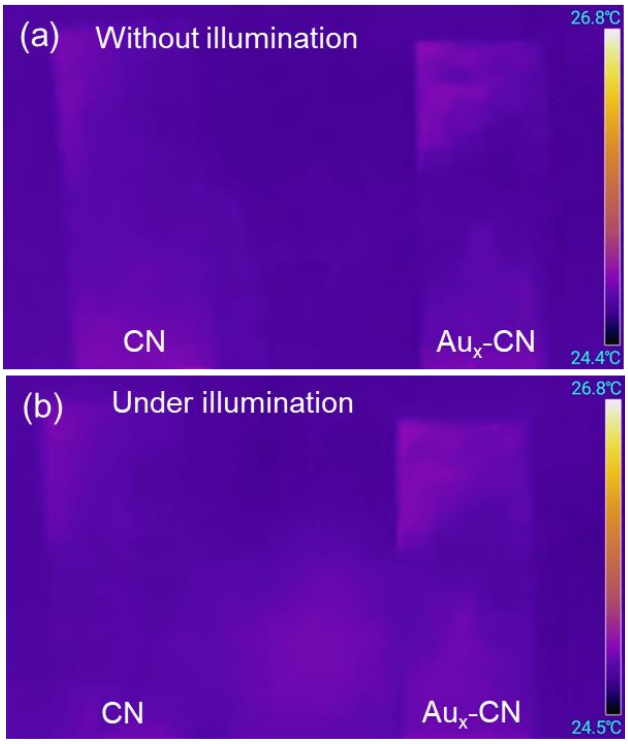

Supplementary Fig. 16. Thermal images of CN and Au_x-CN photoelectrodes showing the photothermal effect without (a) or with (b) irradiation time for 10 min.

It was found that no obvious temperature variations of two photoelectrodes after 10 min irradiation (150 W Xe lamp, Supplementary Fig. 15). And no obvious plasma resonance effect for Au_x-CN photoelectrode was obtained from the UV-vis spectra (Supplementary Fig. 7b). Thus, the photothermal effect and plasma resonance effect of Au species in Au_x-CN were negligible.

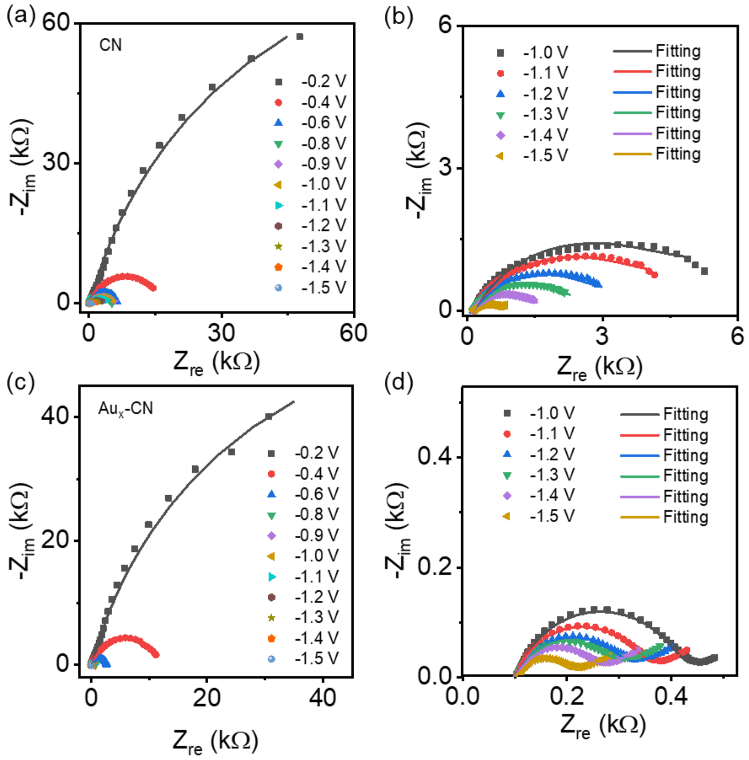

Supplementary Fig. 17 Nyquist plots of (a) the CN and (c) Au_x-CN photoelectrodes at different applied potentials versus Ag/AgCl in 0.01 M phosphate buffer saline containing 0.1 M KCl and 25 mM $K_2S_2O_8$. (b) the corresponding magnification of CN in (a). (d) the corresponding magnification of Au_x-CN in (c). Globules were test data points and solid lines were the fitting data.

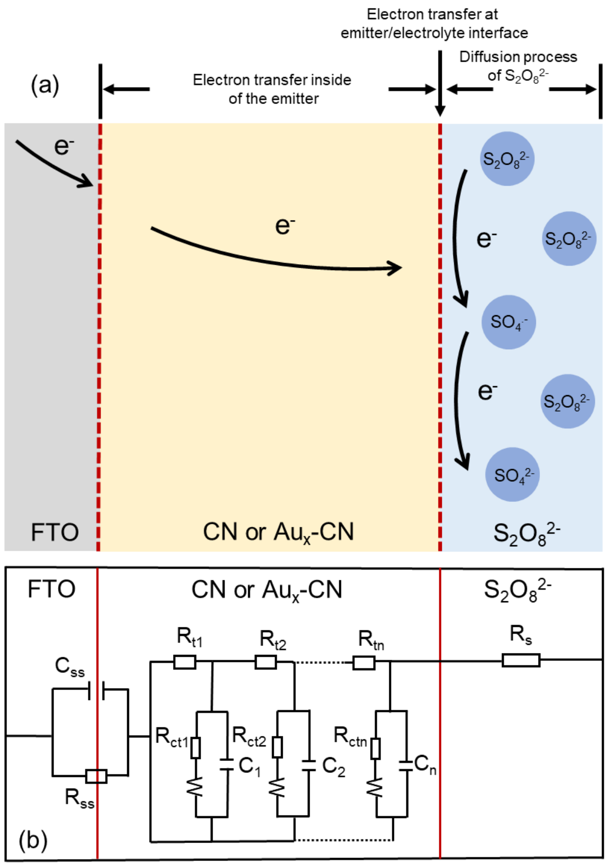

Supplementary Fig. 18 (a) Possible charge transfer processes of ECL in Au_x-CN. (b) Proposed full equivalent circuit used for interpretation of CN and Au_x-CN photoelectrodes.

The R_{ss} and C_{ss} represented contact resistance and capacitance at the interface of the ECL emitter film and FTO. R_{tn}, the series resistance of the film, represented the resistivity of electron transport in the emitter film, which was the intrinsic resistance of the electrode materials. C_n was the double-layer capacitance. R_{ctn} was the charge reaction resistance at the ECL emitter/$S_2O_8^{2-}$ interface, which was caused by the Faradaic reaction. The intimate contact of the CN film and FTO at the nanoscale level allowed us to ignore the first section of the parallel circuit in the simplified equivalent circuit (Fig. 3d, inset). Additionally, the resistances of R_{tn} and R_{ctn} can be combined into the total resistance R_t and R_{ct}.

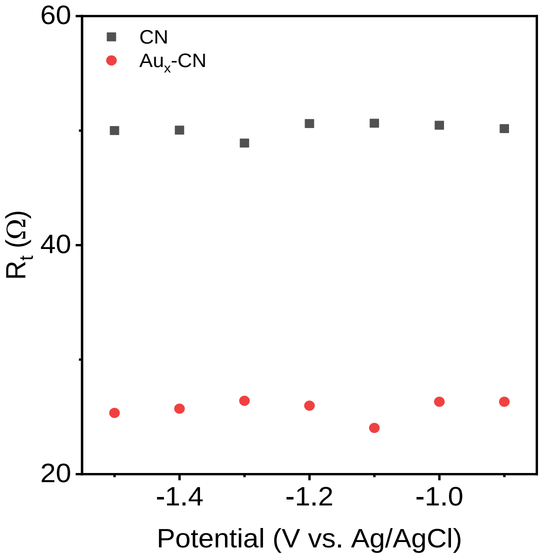

Supplementary Fig. 19 Fitted data for R_t at different overpotentials (-0.9 V~ -1.5 V).

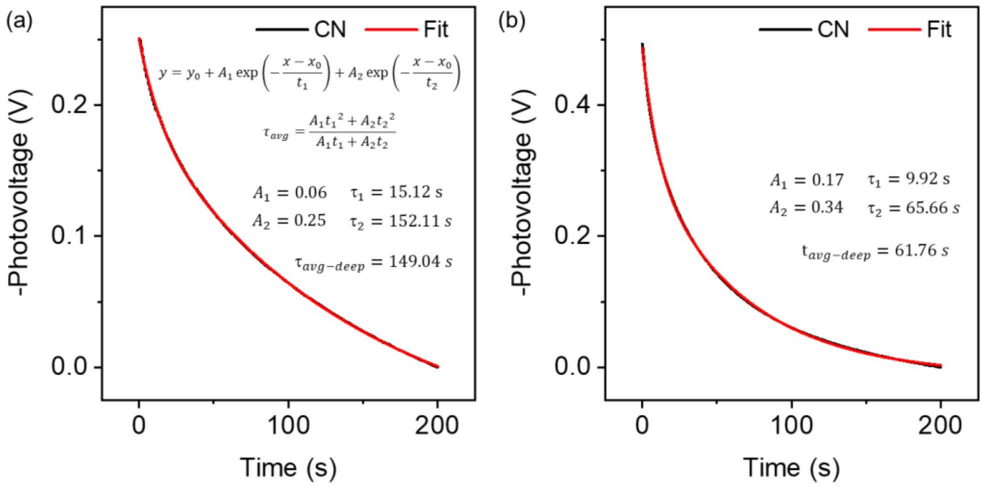

Supplementary Fig. 20 OCP decay curves (black) and fitted analysis (red) of (a) CN and (b) Au_x-CN photoelectrodes.

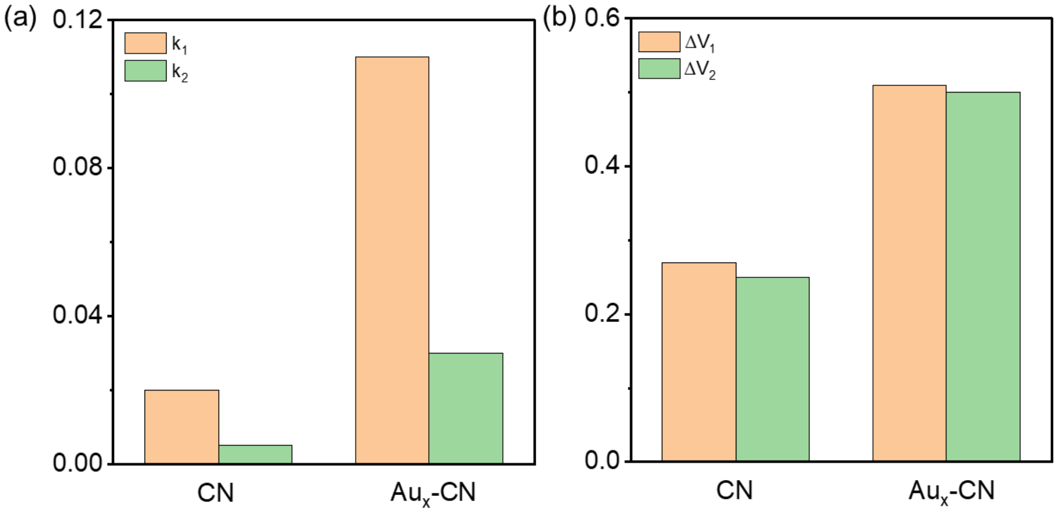

Supplementary Fig. 21 Value of (a) k_1, k_2 and (b) ΔV_1, ΔV_2 for CN and Au_x-CN photoelectrodes obtained from OCP.

Generally, a larger OCP upon irradiation (ΔV_1) is often associated with the more vital excited electron storage ability. It was found that the OCP of two photoelectrodes increased with time under the irradiation and gradually reached a plateau. The slopes of photovoltage drop (k_1) and rise (k_2) after light on and off can be used to characterize the electrochemical excitation and electron-hole recombination kinetics. The larger k_1, k_2 and ΔV_1 for Au_x-CN photoelectrode were observed, indicating the unobstructed electron transfer channel and higher excited electron storage, which provided the origin of the less deep surface electron-trapping state of Au_x-CN photoelectrodes[2].

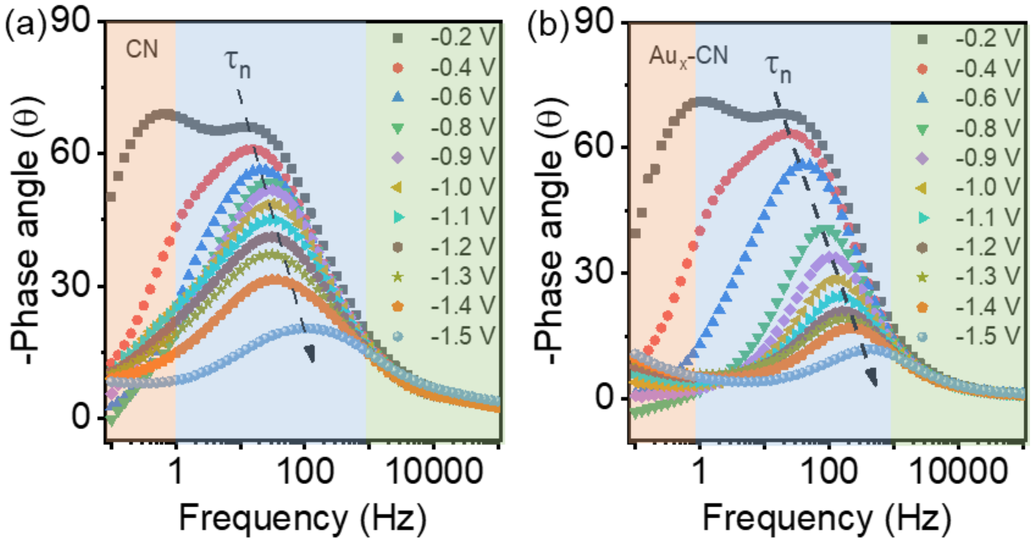

Supplementary Fig. 22 Bode phase plots of (a) CN and (b) Au_x-CN photoelectrodes at different potentials in 0.01 M phosphate buffer saline containing 0.1 M KCl and 25 mM $K_2S_2O_8$.

The Bode plots can be divided into three regions including the high-frequency area (green), mediate frequency area (blue) and low-frequency area (orange). According to previous works, the signal in the high-frequency, mediate frequency and low-frequency region can be ascribed to the solution resistance or intrinsic resistance of the active electrode materials, electron reaction resistance of emitter/electrolyte and Warburg impedance related to the diffusion of $S_2O_8^{2-}$ ions[4], respectively. As shown in Supplementary Fig. 22, phase angle gradually decreased in mediate frequency both for CN and Au_x-CN photoelectrode indicating that the charge-transfer impedance became smaller and the τ_n became shorter with overpotential increasing[5]. Hereinto, the Bode plots in Supplementary Fig. 22 (-1.5 V, i.e., Fig. 3h) showed the lower phase angle in high and mediate frequency area for Au_x-CN photoelectrode than that of CN photoelectrode. It indicated that the carrier diffusion dynamics in bulk emitter and electron transfer kinetic at the emitter/$S_2O_8^{2-}$ interface became faster after Au dropping[4].

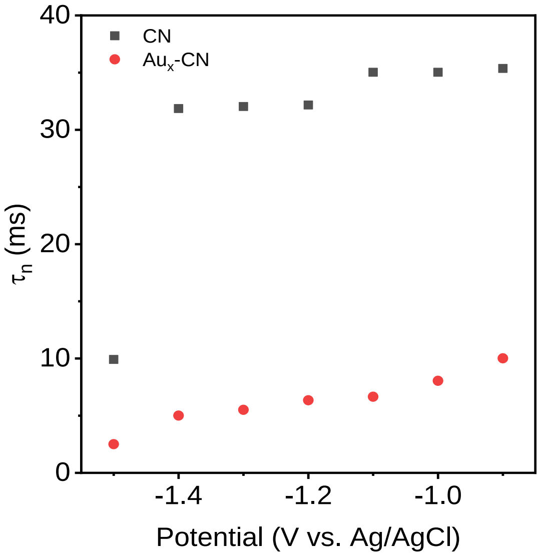

Supplementary Fig. 23 Bias dependence of characteristic lifetime τ_n extracted from mediate frequency EIS spectra of CN and Au_x-CN photoelectrodes.

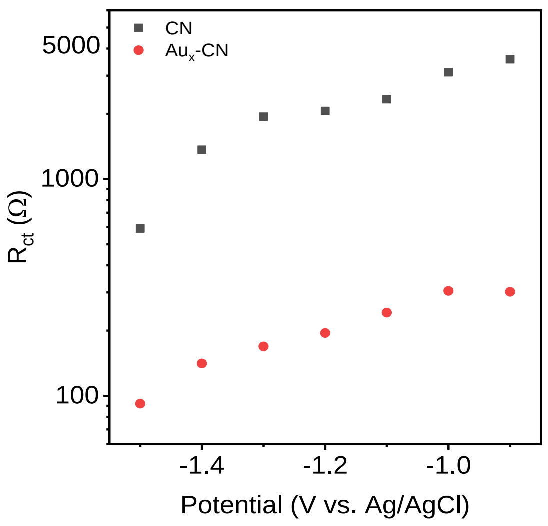

Supplementary Fig. 24 Fitted data for R_{ct} at different overpotentials (-0.2 V~ -1.5 V) of CN and Au_x-CN photoelectrodes.

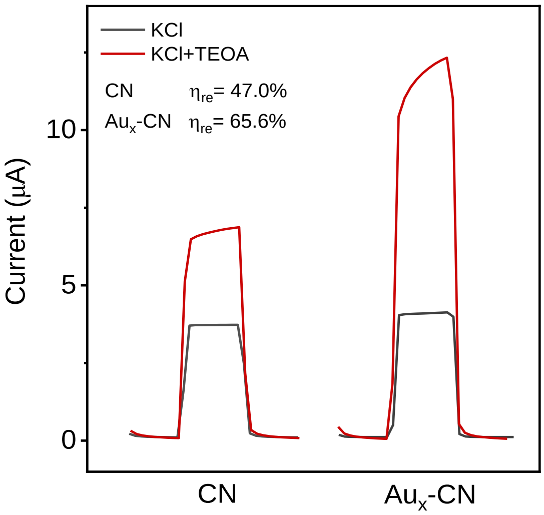

Supplementary Fig. 25 Electron-hole recombination efficiency of CN and Au_x-CN photoelectrodes evaluated by PEC current under chopped light biased at -0.3 V vs. Ag/AgCl in 0.1 M KCl.

Triethanolamine (TEOA), an efficient hole scavenger, was added to the electrolytes. Assuming that most holes are successfully extracted in the system, the normalized photocurrent after the addition of TEOA could be set as 100%[6]. Based on this speculation, the calculated electron-hole recombination efficiency (η_{re}, Eq. 6) of Au_x-CN photoelectrode was higher, supporting the as-observed lower TAS intensity (Fig. 3e).

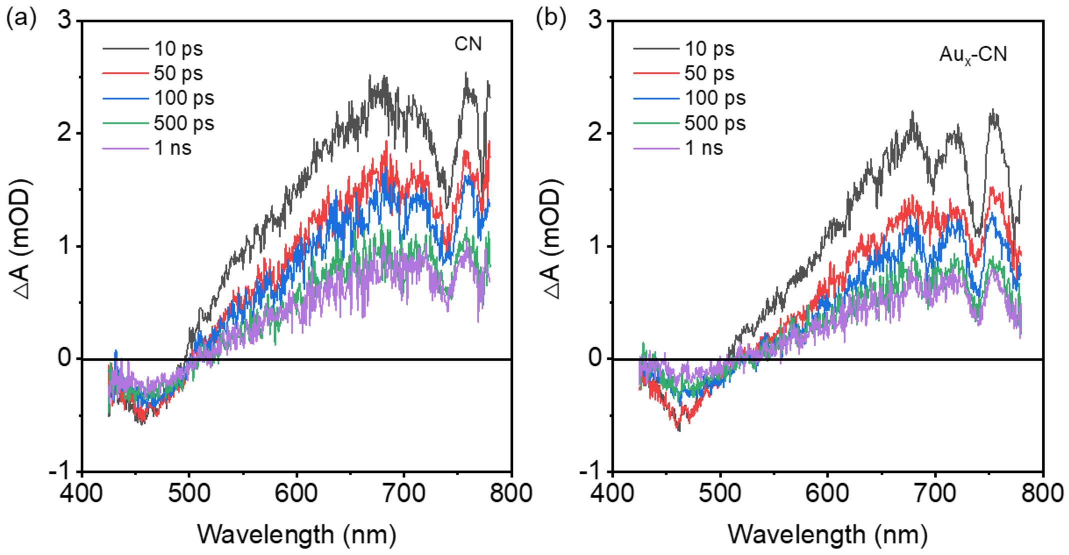

Supplementary Fig. 26 Visible femtosecond transient absorption spectra of (a) CN and (b) Au_x-CN photoelectrodes at selected time points from 10 ps to 1 ns.

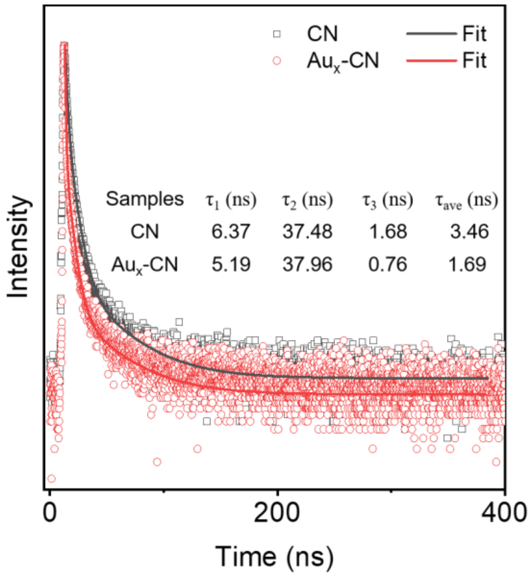

Supplementary Fig. 27 Time-resolved FL decay spectra of CN and Au_x-CN photoelectrodes under 365 nm excitation.

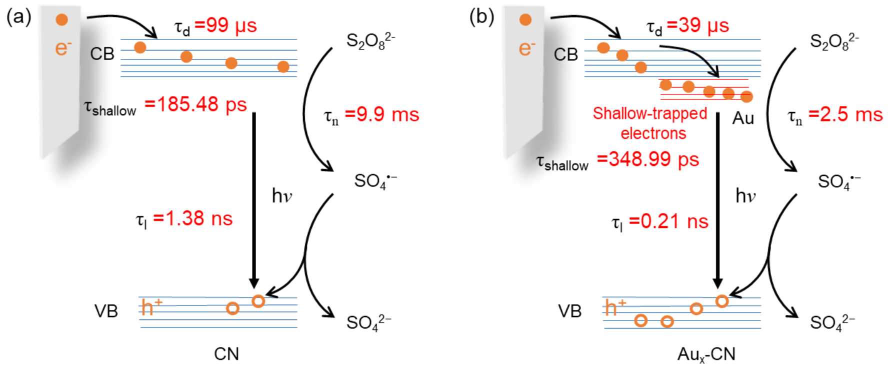

Supplementary Fig. 28 Possible mechanism for ECL of (a) CN photoelectrode and (b) Au_x-CN photoelectrode with different timescale.

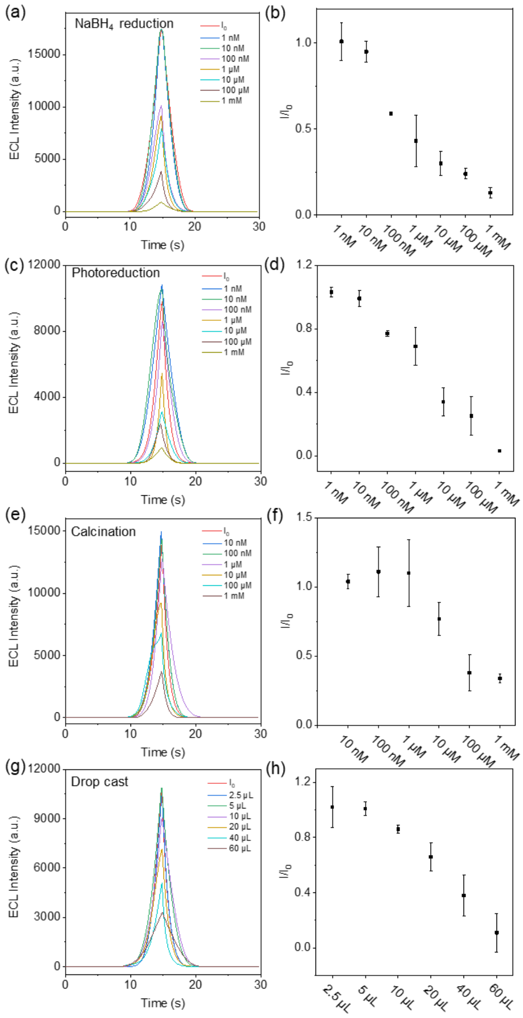

Supplementary Fig. 29 ECL emission intensity of different control Au/CN photoelectrodes made by (a) $NaBH_4$ reduction, (c) photoreduction, (e) calcination, and (g) drop cast methods. ECL emission intensity change value (I/I_0) of different control Au/CN photoelectrodes made by (b) $NaBH_4$ reduction, (d) photoreduction, (f) calcination, and (h) drop cast methods. I_0 and I were the ECL intensity before and after the addition of Au species, respectively. Error bars represent the standard error derived from three independent measurements. "a.u." refers to arbitrary units.

To examine the impact of adsorption on the ECL system, the control Au/CN photoelectrodes (Au species on the surface of the CN photoelectrode) were synthesized using most common methods including calcination, drop casting, sodium borohydride ($NaBH_4$) reduction, and photoreduction. As shown in Supplementary Fig. 29, the ECL emission intensity of the Au/CN photoelectrodes, varying in Au content, did not demonstrate evident enhancement. It indicated that the Au species did not exhibit a noticeable adsorption effect on the $S_2O_8^{2-}$ in this study.

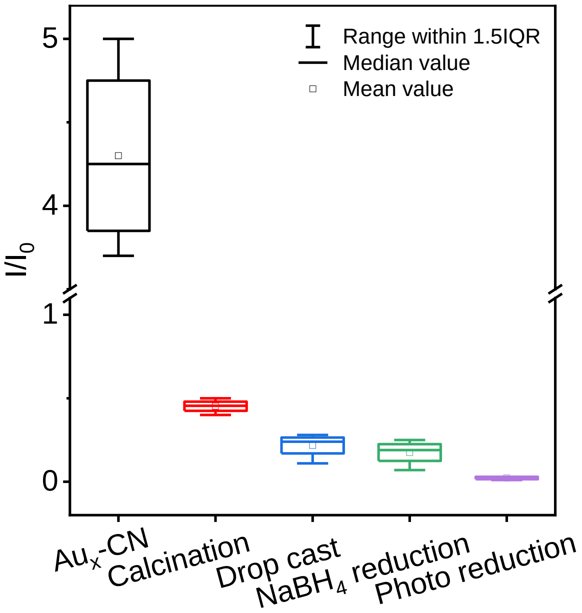

Supplementary Fig. 30 ECL emission intensity change (I/I_0) of Au_x-CN photoelectrode and various control Au/CN photoelectrodes. I_0 and I were the ECL intensity before and after the addition of Au species, respectively. The boxplots display the interquartile range (boxes), the median (horizontal lines in the middle), the 25th and 75th percentile (horizontal lines in the bottom and top) and the mean (square), $n = 4$ per group.

As shown in Supplementary Fig. 30, the ECL intensity of various control Au/CN photoelectrodes was quenching, and only the ECL intensity of Au_x-CN photoelectrode was enhanced.

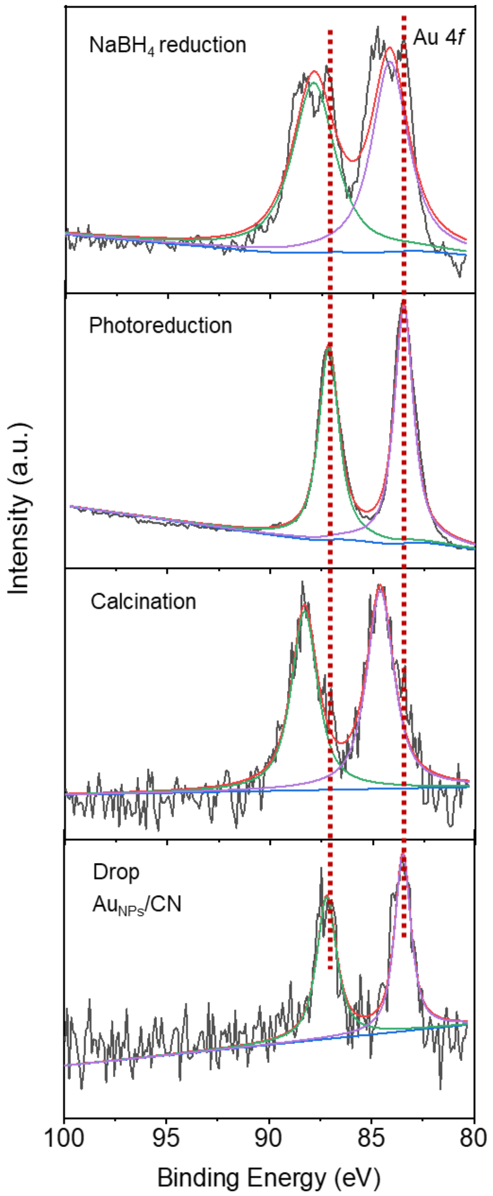

Supplementary Fig. 31 High-resolution Au 4*f* XPS spectra of control Au/CN photoelectrodes. "a.u." refers to arbitrary units.

The high-resolution Au 4*f* XPS spectra were shown in Supplementary Fig. 31. For Au_{NPs}/CN, the peaks at 86.83 and 83.07 eV can be attributed to the metallic Au^0 $4f^{5/2}$ and Au^0 $4f^{7/2}$, respectively. More importantly, compared with those of Au_{NPs}/CN counterparts, the Au $4f^{5/2}$ and Au $4f^{7/2}$ peaks of Au/CN both shifted to higher binding energies. The positive position of binding energy indicated that the Au surface in Au_{NPs}/CN was oxidation state and metallic state. All of the control Au/CN photoelectrodes had the Au-N bond according to the literature[7-9].

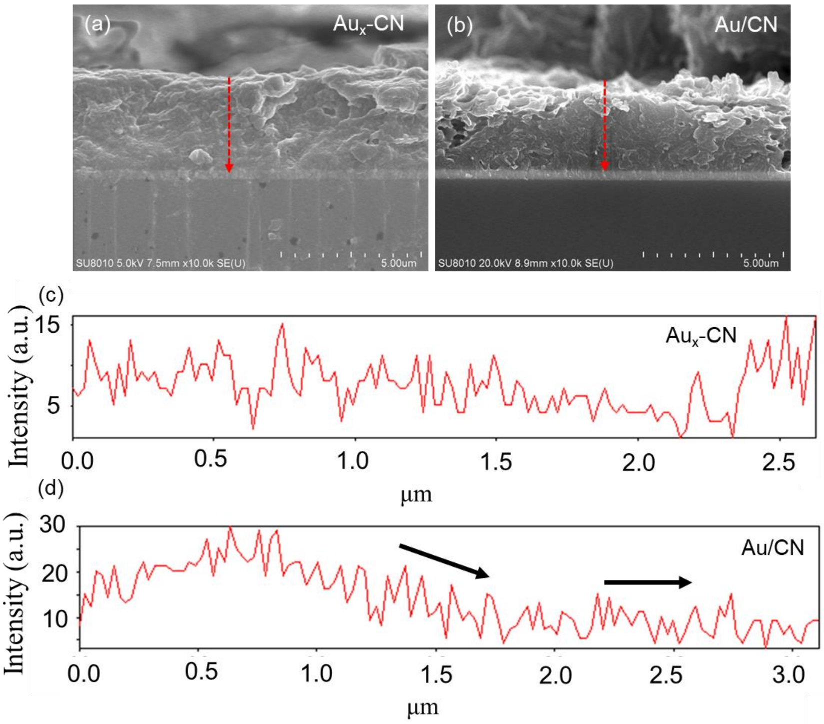

Supplementary Fig. 32 Top-sectional SEM images of (a) Au$_x$-CN and (b) Au/CN photoelectrodes. The corresponding cross-section SEM-EDS linear scans of (c) Au$_x$-CN and (d)Au/CN photoelectrodes. “a.u.” refers to arbitrary units.

In order to investigate the distribution of the Au element, the SEM (Supplementary Figs. 32a and b) and cross-section SEM-EDS linear scans were conducted (Supplementary Figs. 32c and d). The semi-quantitative atomic Au content obtained from cross-section SEM-EDS linear scans of the Au$_x$-CN photoelectrode in the perpendicular direction almost did not show significant changes because the Au species were uniformly distributed between layers of CN (Supplementary Fig. 32c). However, the Au content demonstrated a sharp increase at the top of the Au/CN photoelectrode in the perpendicular direction, indicating the amount of Au species on the top of the Au/CN photoelectrode (Supplementary Fig. 32d).

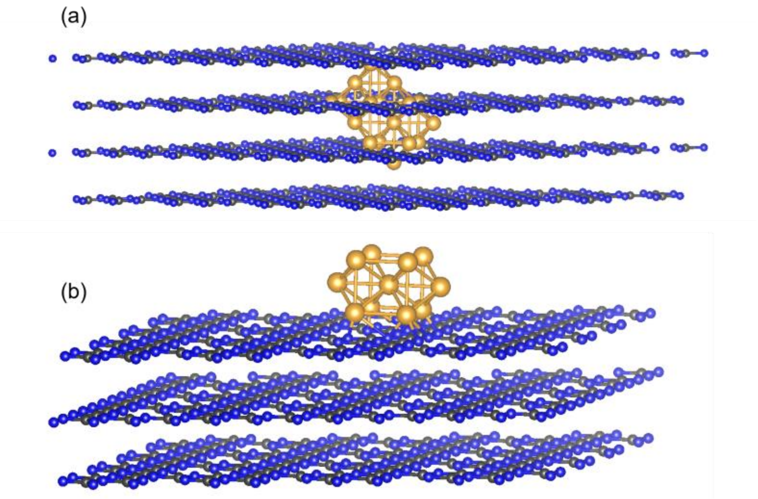

Supplementary Fig. 33 Structure model of (a) Au_x-CN and (b) various Au/CN. Gray coloring indicates carbon atoms, blue indicates nitrogen, and orange indicates gold.

Au-N position. According to the SEM images and EDS analysis (Supplementary Fig. 32), the corresponding structure models of Au_x-CN and Au/CN photoelectrode were shown in Supplementary Fig. 33. The Au species were presented in the interlayer of CN for Au_x-CN photoelectrode, but for Au/CN photoelectrode, they were presented on the top of the CN surface. Combined with the quenching phenomenon of ECL intensity for Au/CN photoelectrodes, we proved that the ECL emission enhancement of CN was attributed to the interlayer Au-N bond instead of the top surface of CN.

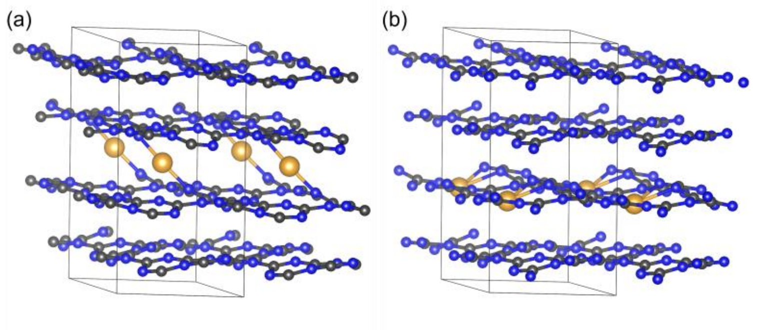

Supplementary Fig. 34 Two possible simplified Au_x-CN models. (a) Au atoms were bonded between two layers of CN, and (b) Au atoms were inserted into the hole in a CN layer. Gray coloring indicates carbon atoms, blue indicates nitrogen, and orange indicates gold.

Compared to the electron transfer mode in pristine CN, the electron transfer between Au species and CN layers in Au_x-CN was the vital step. In order to reduce computational complexity, two simplified Au_x-CN models were shown in Supplementary Fig. 32. The Au-N bonds between the Au atoms and the two adjacent CN layers were 2.06 Å and 2.07 Å (Supplementary Fig. 32a), respectively, which were close to the EXAFS results. Another possible structure (Supplementary Fig. 32b) in which the Au atoms located in the intra-layer holes of CN and the distances between Au-N were all larger than 2.39 Å (2.39 Å, 2.40 Å, 2.49 Å, 2.51 Å, 2.59 Å), which was inconsistent with the experimental results. Therefore, the influence of Au atoms on the inter-layer charge transfer was primarily explored in the DFT calculation.

It should be noted that both Au nanoparticles and single atoms were observed in the Au_x-CN photoelectrode. The DFT calculation in Supplementary Fig. 33a and Supplementary Fig. 34 showed both Au nanoparticles and single atoms formed Au-N bonds between the Au atoms and the two adjacent CN layers. Quantitative ECL kinetics measurements and theoretic calculations jointly disclosed Au-N bonds endowed shallow trapped electron states, which coordinated the timescale of the fast electron transfer in the bulk emitter and the slow redox reaction of co-reagent at diffusion layers. Besides, more and more research reported that the charge transfer between nanoparticles and single atoms enables to modulation of the electronic structure of materials, resulting in enhanced performance.[10,11]

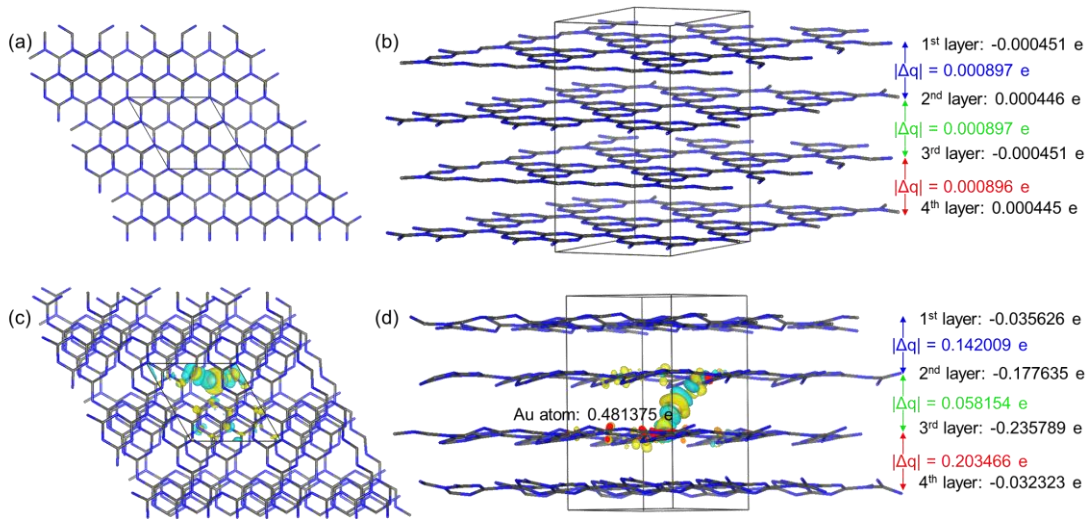

Supplementary Fig. 35 Interlayer charge transfer in Au_x-CN. Charge distribution analysis of (a, b) CN and (c, d) Au_x-CN. |Δq| represents the absolute value of the difference in the electron distribution between the layers. The stick model describes CN, gray coloring indicates carbon atoms, blue indicates nitrogen, and orange indicates gold. Yellow and blue represent electron accumulation and electron depletion, respectively.

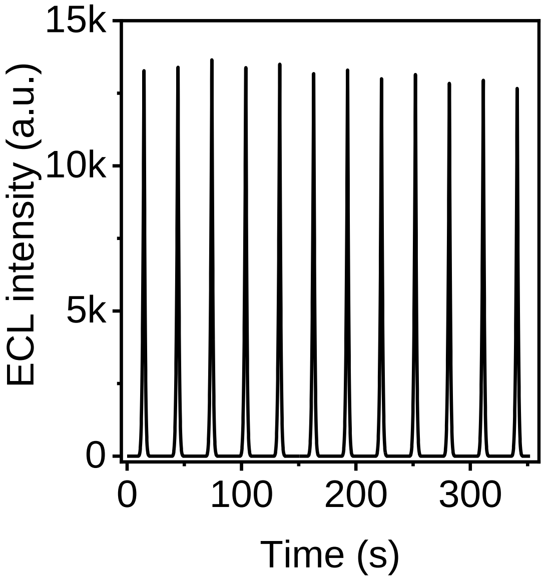

Supplementary Fig. 36 ECL intensity of the as-proposed biosensor for detection of NO_2^- under consecutive cyclic potential scanning. "a.u." refers to arbitrary units.

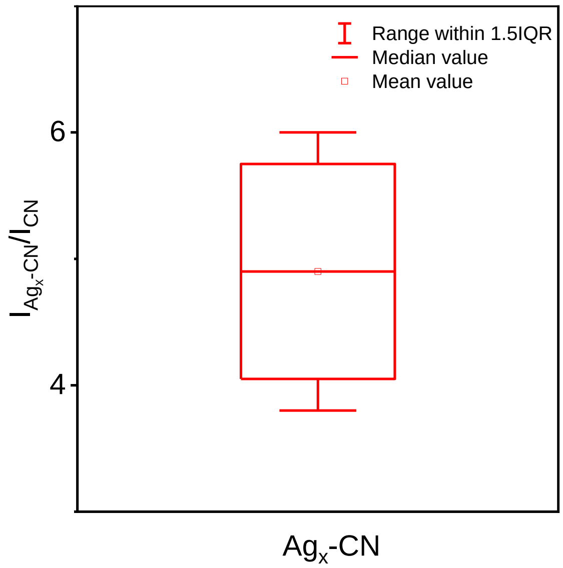

Supplementary Fig. 37 Ratio of ECL intensity for Ag_x-CN to CN photoelectrode. The boxplots display the interquartile range (boxes), the median (horizontal lines in the middle), the 25th and 75th percentile (horizontal lines in the bottom and top) and the mean (square), $n = 4$ per group.

Supplementary Table 1 Comparison of the relative co-reactant ECL efficiency of different lumiphores by considering the number of generated photons and consumed electrons (Eq. 7).

Luminphores	ECL	Standard (100%)	Φ_{ECL}	ref
silole-containing polymer	anodic	$Ru(bpy)_3^{2+}$/TPrA	39[a]	12
Au NCs/Cu_2O	anodic	$Ru(bpy)_3^{2+}$/TEA	0.64[b]	13
iridium(III) complex ($(bt)_2$Irbza)	anodic	$Ru(bpy)_3^{2+}$/TPrA	1670[b]	14
H_4TCBPE	anodic	$Ru(bpy)_3^{2+}$/TPrA	0.22[b]	15
$(tmeda)_6Zn_8Mn_6Se_{13}C_{l2}$	cathodic	$[Ru(bpy)_3](PF_6)_2$/ $S_2O_8^{2-}$	0.3[a]	16
TBPE-CMPs	anodic	$Ru(bpy)_3^{2+}$/TPrA	0.02[a]	17
Pdots	anodic	$Ru(bpy)_3^{2+}$/TEA	400[a]	18
$(tmeda)_6Zn_{14}Se_{13}Cl_2$	cathodic	$[Ru(bpy)_3](PF_6)_2$/ $S_2O_8^{2-}$	0.3[a]	16
N,S-doped graphene quantum dots	cathodic	$Ru(bpy)_3^{2+}/S_2O_8^{2-}$	32[a]	19
BODIPY-Capped Nanocrystals	anodic	$Ru(bpy)_3^{2+}$/TPrA	96[a]	16
polymer dots	anodic	$Ru(bpy)_3^{2+}$/TPrA	23.1[a]	20
polymer dots	anodic	$Ru(bpy)_3^{2+}$/TEA	400[a]	20
$[(bpy)_2Ru]_2(bphb)^{4+}$	annihilation	$Ru(bpy)_3^{2+}$ (50:50 (v/v) MeCN/H_2O)	200-300[a]	21
$[(bpy)_2Ru]_2(bphb)^{4+}$	cathodic	$Ru(bpy)_3^{2+}/S_2O_8^{2-}$(acetonitrile (MeCN), 50:50 (v/v) MeCN/H_2O)	60-80[a]	21
$[(dFphtl)_2Ir(dma1bpydma_2)]^+$	annihilation	$Ru(bpy)_3^{2+}$	550[a]	22
iridium(III)-compounds	cathodic	$Ru(bpy)_3^{2+}$/BPO	103[a]	23
CdSe/CdS/ZnS core/shell/shell QDs	cathodic	$Ru(bpy)_3^{2+}/S_2O_8^{2-}$	69000000[b]	24
CN-NV-550	cathodic	$Ru(bpy)_3^{2+}/S_2O_8^{2-}$	17900[b]	25
Ox-Met-AuNCs	anodic	$Ru(bpy)_3^{2+}$/TEA	66.1[b]	23

Vacancy@CdInS	anodic	$Ru(bpy)_3^{2+}$/TPrA	2.1[b]	26
Vacancy@CdInS	cathodic	$Ru(bpy)_3^{2+}/S_2O_8^{2-}$	0.13[b]	26
Mn@CdInS	anodic	$Ru(bpy)_3^{2+}$/TPrA	0.8[b]	26
Mn@CdInS	cathodic	$Ru(bpy)_3^{2+}/S_2O_8^{2-}$	0.036[b]	26
$Au_{12}Ag_{13}$ nano-clusters	annihilation	$Ru(bpy)_3^{2+}$	1000[b]	27
$Au_{12}Ag_{13}$ nano-clusters	anodic	$Ru(bpy)_3^{2+}$/TPrA	40000[b]	27
ATT-AuNCs	anodic	$Ru(bpy)_3^{2+}$/TEA	78[b]	27
NHCDs	anodic	$Ru(bpy)_3^{2+}/H_2O_2$	250[b]	28
Pt-PEG_2	anodic	$Ru(bpy)_3^{2+}$/TPrA	120[b]	29
Au-LA-DEDA	anodic	$Ru(bpy)_3^{2+}$/TPrA	170[b]	30
CdInS NCs	annihilation	$Ru(bpy)_3^{2+}$	21.72[b]	31
PEI@Ru-PCN-777	annihilation	$Ru(bpy)_3^{2+}$	6.0[b]	32
PEI@Ru-Hf-MOL	annihilation	$Ru(bpy)_3^{2+}$	7.6[b]	32
Polydopamine ECL-organic nanoparticles	cathodic	$Ru(bpy)_3^{2+}/S_2O_8^{2-}$	2.9[b]	33
5′-QSA	cathodic	$Ru(bpy)_3^{2+}/S_2O_8^{2-}$	29[c]	34
Nafion-$Ru(bpy)_3]^{2+}/Na_2C_2O_4$	anodic	$Ru(bpy)_3^{2+}$(1 mM and 0.1 M $(TBA)BF_4$/acetonitrile)	1.78[c]	35
$[Ru(bpy)_2(PVP)_{10}]^{2+}/Na_2C_2O_4$	anodic	$Ru(bpy)_3^{2+}$(1 mM and 0.1 M $(TBA)BF_4$/acetonitrile)	3.04[c]	35
Hollow Porous Polymeric Nanospheres of a Self-Enhanced Ruthenium Complex	anodic	$[Ru(bpy)_3]^{2+}$ (1 mM and 0.1 M $(TBA)BF_6$/acetonitrile)	7.2[c]	36
$tPDI_2N$-TMB/TPrA	anodic	$Ru(bpy)_3^{2+}$/TPrA	1.65[b]	37
NSF-CDs	cathodic	$Ru(bpy)_3^{2+}/S_2O_8^{2-}$	4.49[b]	38
CN_{MW}	cathodic	$Ru(bpy)_3^{2+}/S_2O_8^{2-}$	700[a]	3

CN_{MW580}	cathodic	$Ru(bpy)_3^{2+}/S_2O_8^{2-}$	17000[a]	2
CN_{MW580}	cathodic	$Ru(bpy)_3^{2+}/S_2O_8^{2-}$	540452[b]	2
CN-410	cathodic	$Ru(bpy)_3^{2+}/S_2O_8^{2-}$	11176[a]	39
CN-450	cathodic	$Ru(bpy)_3^{2+}/S_2O_8^{2-}$	39354[a]	39
CN-470	cathodic	$Ru(bpy)_3^{2+}/S_2O_8^{2-}$	35289[a]	39
CN-525	cathodic	$Ru(bpy)_3^{2+}/S_2O_8^{2-}$	25067[a]	39
tpCN	cathodic	$Ru(bpy)_3^{2+}/S_2O_8^{2-}$	2256[a]	40
Au_x-CN	cathodic	$Ru(bpy)_3^{2+}/S_2O_8^{2-}$	3261[a]	This work

The number of photons for Φ_{ECL} calculation was derived from [a] wavelength-resolved ECL spectrum, [b] ECL intensity, or [c] methods not available (N/A).

Supplementary Table 2 Impedance fitting data for FTO, CN and Au_x-CN photoelectrode in 5 mM $[Fe(CN)_6]^{3-}/[Fe(CN)_6]^{4-}$ and 0.1 M KCl at open circuit potential (-0.23 V).

	R_u (ohm)	Y_0 ($S*s^a$)	α	W_d ($S*s^{1/2}$)	R_{ct} (ohm)
FTO	173.3	$5.2e^{-6}$	0.9	0.01	60.58
CN	229	$1.3e^{-6}$	0.8	25.37	$1.23e^{5}$
Au_x-CN	237.7	$1.0e^{-6}$	0.8	0.8	$7.88e^{5}$

R_u: solution resistance; Y_0: capacitance; α: constant phase; W_d: Warburg resistance; R_{ct}: interfacial charge transfer resistance across the electrode/electrolyte.

Supplementary Table 3 Summary of the impedance fitting data for CN photoelectrode in 0.01 M phosphate buffer saline containing 0.1 M KCl and 25 mM $K_2S_2O_8$ at different potential.

Potential	R_u (ohm)	Y_1 ($S*s^a$)	α_1	R_{ct} (ohm)	R_t (ohm)	Y_2 ($S*s^a$)	α_2
-0.2 V	80.60	$1.94e^{-5}$	0.81	231100	63.25	$1.11e^{-5}$	$5.03e^{-7}$
-0.4 V	81.90	$2.43e^{-5}$	0.77	11520	56.26	$2.0e^{-4}$	$1.80e^{-6}$
-0.6 V	82.32	$2.41e^{-5}$	0.77	6441	55.51	$9.23e^{-3}$	$4.32e^{-5}$
-0.8 V	80.10	$2.57e^{-5}$	0.75	4499	54.11	$2.17e^{-3}$	$7.21e^{-4}$
-0.9 V	81.05	$2.58e^{-5}$	0.74	3568	50.17	$5.71e^{-4}$	0.22
-1.0 V	82.21	$3.60e^{-5}$	0.71	3109	50.46	$4.88e^{-4}$	0.20
-1.1 V	80.09	$4.66e^{-5}$	0.69	2335	50.64	$5.21e^{-4}$	0.15
-1.2 V	79.60	$5.55e^{-5}$	0.67	2060	50.61	$1.04e^{-3}$	0.17
-1.3 V	80.00	$8.52e^{-5}$	0.61	1939	48.91	$2.95e^{-3}$	0.26
-1.4 V	79.80	$9.99e^{-5}$	0.58	1365	50.04	$7.08e^{-3}$	0.39
-1.5 V	80.00	$1.18e^{-4}$	0.52	591	50.00	$6.65e^{-3}$	0.38

R_u: solution resistance; Y_1: capacitance associated with constant phase element (CPE1); α_1: constant phase associated with CPE1; R_{ct}: interfacial charge transfer resistance across the electrode/electrolyte; R_t: electron transport resistance; Y_2: capacitance associated with constant phase element (CPE2); α_2: constant phase associated with CPE2.

Supplementary Table 4 Summary of the impedance fitting data for Au_x-CN photoelectrode in 0.01 M phosphate buffer saline containing 0.1 M KCl and 25 mM $K_2S_2O_8$ at different potential.

Potential	Ru (ohm)	Y_1 ($S*s^a$)	α_1	R_{ct} (ohm)	R_t (ohm)	Y_2 ($S*s^a$)	α_2
-0.2 V	82.50	$1.79e^{-5}$	0.83	121200	23.36	$1.46e^{-3}$	$1.41e^{-5}$
-0.4 V	81.50	$2.20e^{-5}$	0.83	4411	24.00	$1.41e^{-3}$	$2.5e^{-2}$
-0.6 V	78.79	$1.91e^{-5}$	0.85	1713	26.08	$1.12e^{-3}$	$2.7e^{-2}$
-0.8 V	79.28	$1.79e^{-5}$	0.86	653.3	26.04	$9.40e^{-2}$	0.19
-0.9 V	78.40	$1.82e^{-5}$	0.82	302	26.32	$1.42e^{-2}$	0.24
-1.0 V	78.40	$1.82e^{-5}$	0.82	305	26.32	$1.48e^{-2}$	0.25
-1.1 V	80.20	$2.40e^{-5}$	0.79	242	24.03	$1.29e^{-2}$	0.32
-1.2 V	80.10	$3.30e^{-5}$	0.76	195	25.98	$1.02e^{-2}$	0.30
-1.3 V	79.63	$3.20e^{-5}$	0.77	169	26.4	$8.85e^{-3}$	0.29
-1.4 V	80.79	$2.57e^{-5}$	0.76	141	25.71	$1.00e^{-2}$	0.25
-1.5 V	80.90	$1.51e^{-5}$	0.72	92	25.34	$1.10e^{-2}$	0.26

R_u: solution resistance; Y_1: capacitance associated with constant phase element (CPE1); α_1: constant phase associated with CPE1; R_{ct}: interfacial charge transfer resistance across the electrode/electrolyte; R_t: electron transport resistance; Y_2: capacitance associated with constant phase element (CPE2); α_2: constant phase associated with CPE2.

Supplementary Table 5 Comparison of the NO_2^- sensing performance.

Methods	Materials	Linear range	LOD	Ref
UV-Vis	Au NPs	1-5 pM	1 pM	41
FL	Carbon quantum dots	0.05-1.0 μM<br>1.0-50 μM	7.1 nM	42
Electrochemistry	TiO_2	0.5-10 mg L^{-1}	0.2 mg L^{-1}	43
Electrochemistry	TiO_2	0.1-5 mg L^{-1}	0.06 mg L^{-1}	43
PEC	graphene – TiO_2 nanowire	0.5-9000 mM	0.225 mM	44
Electrochemistry	MWCNTs–TiN/Cyt c	1.0-2000 μM	1.4 nM	45
Electrochemistry	Mb/Au-PTy-f-MWCNTs	1.0-8000 μM	2 nM	46
FL	Benzimidazole	10-100 μM	21.7 μM	47
UV-Vis	AuNPs@polymyxin	0.2-2 μM	0.18 μM	48
Electrochemistry	ZrO_2@MWCNTs	5-100 μM	0.94 μM	49
UV-Vis	4-ATP modified GNR	5.2−100 μM	1 pM	50
FL	Rh 6G-SiO_2	-	1.2 μM	51
Electrochemistry	ZnS	20 nM - 1.35 mM	8.5 nM	52
UV-Vis	1-Nap preloaded PEG-hydrogel	-	10 μM	53
ECL	Au_x-CN	10^{-9} to 10^{-15} M	0.21 fM	This work

Multi-walled carbon nanotube: MWCNTs; cytochrome c: Cyt c; Myoglobin: Mb; Poly tyramine: Pty; AuNPs@polymyxin: AuNPs functionalized with polymyxin molecules; ATP: aminothiophenol; GNR: Au nanorods; Rh: rhodamine 6G: p-hydroxybenzaldlehyde rhodamine 6G hydrozone; 1-Nap: *N*-(1-naphthyl)ethylenediamine, PEG: Poly(ethylene glycol) diacrylate.

Supplementary references

1 Huang, C. *et al.* Unraveling Fundamental Active Units in Carbon Nitride for Photocatalytic Oxidation Reactions. *Nat. Commun.* **12**, 320 (2021).

2 Fang, Y. *et al.* Elucidating Orbital Delocalization Effects on Boosting Electrochemiluminescence Efficiency of Carbon Nitrides. *Adv. Opt. Mater.* **10**, 2201017 (2022).

3 Zhao, T. *et al.* Ultrafast Condensation of Carbon Nitride on Electrodes with Exceptional Boosted Photocurrent and Electrochemiluminescence. *Angew. Chem. Int. Ed.* **59**, 1139-1143 (2019).

4 Xie, C. *et al.* In-situ Phase Transition of WO_3 Boosting Electron and Hydrogen Transfer for Enhancing Hydrogen Evolution on Pt. *Nano Energy* **71**, 104653 (2020).

5 Wang, Q., Moser, J.-E. & Grätzel, M. Electrochemical Impedance Spectroscopic Analysis of Dye-Sensitized Solar Cells. *J. Phys. Chem. B* **109**, 14945-14953 (2005).

6 Qin, J. *et al.* Direct Growth of Uniform Carbon Nitride Layers with Extended Optical Absorption towards Efficient Water-Splitting Photoanodes. *Nat. Commun.* **11**, 4701 (2020).

7 Chen, Z. *et al.* Single-Site Au^{I} Catalyst for Silane Oxidation with Water. *Adv. Mater.* **30**, 1704720 (2017).

8 Zhang, L. *et al.* Tuning Metal Catalyst with Metal–C_3N_4 Interaction for Efficient CO_2 Electroreduction. *ACS Catal.* **8**, 11035-11041 (2018).

9 Chen, Z. *et al.* Single-Atom Au^{I}–N_3 Site for Acetylene Hydrochlorination Reaction. *ACS Catal.* **10**, 1865-1870 (2020).

10 Feng, Y. *et al.* Spherical vs. planar: Steering the electronic communication between Ru nanoparticle and single atom to boost the electrocatalytic hydrogen evolution activity both in acid and alkaline. *Applied Catalysis B: Environmental* **307**, 121193 (2022).

11 Wu, Y., Tang, X., Yuan, K. & Chen, Y. Single-atom sites combined with metal nano-aggregates for efficient electrocatalysis. *Energy Environ. Sci.* **16**, 5663-5687 (2023).

12 Feng, Y., Dai, C., Lei, J., Ju, H. & Cheng, Y. Silole-Containing Polymer Nanodot: An Aqueous Low-Potential Electrochemiluminescence Emitter for Biosensing. *Anal. Chem.* **88**, 845-850 (2015).

13 Zhu, X., Zhang, X., Zhou, Y., Chai, Y. & Yuan, R. High-Efficient Electrochemiluminescence of Au Nanoclusters Induced by the Electrosensitizer Cu_2O: The Mechanism Insights from the Electrogenerated Process. *Anal. Chem.* **93**, 10212-10219 (2021).

14 Xu, Z.-H. *et al.* Ultrasensitive Nucleic Acid Assay Based on Cyclometalated Iridium(III) Complex with High Electrochemiluminescence Efficiency. *Anal. Chem.* **93**, 1686-1692 (2020).

15 Huang, W. *et al.* Matrix Coordination-Induced Electrochemiluminescence Enhancement of Tetraphenylethylene-Based Hafnium Metal–Organic Framework: An Electrochemiluminescence Chromophore for Ultrasensitive

Electrochemiluminescence Sensor Construction. *Anal. Chem.* **92**, 3380-3387 (2020).

16 Hesari, M. *et al.* Enhancing Electrochemiluminescence of Chalcogenide Clusters by Means of Mn Replacement. *Electrochim Acta* **210**, 79-86 (2016).

17 Wang, Q. *et al.* Ternary Electrochemiluminescence Biosensor Based on DNA Walkers and AuPd Nanomaterials as a Coreaction Accelerator for the Detection of miRNA-141. *ACS Appl. Mater. Interfaces* **13**, 25783-25791 (2021).

18 Wang, N. *et al.* Dual Intramolecular Electron Transfer for In Situ Coreactant-Embedded Electrochemiluminescence Microimaging of Membrane Protein. *Angew. Chem. Int. Ed.* **60**, 197-201 (2020).

19 Zhang, R., Adsetts, J. R., Nie, Y., Sun, X. & Ding, Z. Electrochemiluminescence of Nitrogen- and Sulfur-doped Graphene Quantum Dots. *Carbon* **129**, 45-53 (2018).

20 Wang, N. *et al.* Dual Resonance Energy Transfer in Triple-component Polymer Dots to Enhance Electrochemiluminescence for Highly Sensitive Bioanalysis. *Chem Sci* **10**, 6815-6820 (2019).

21 Richter, M. M., Bard, A. J., Kim, W. & Schmehl, R. H. Electrogenerated Chemiluminescence. 62. Enhanced ECL in Bimetallic Assemblies with Ligands That Bridge Isolated Chromophores. *Anal. Chem.* **70**, 310-318 (1998).

22 Swanick, K. N., Ladouceur, S., Zysman-Colman, E. & Ding, Z. Self-Enhanced Electrochemiluminescence of an Iridium(III) Complex: Mechanistic Insight. *Angew. Chem. Int. Ed.* **124**, 11241-11244 (2012).

23 Peng, H. *et al.* Pre-oxidation of Gold Nanoclusters Results in a 66 % Anodic Electrochemiluminescence Yield and Drives Mechanistic Insights. *Angew. Chem. Int. Ed.* **131**, 11817-11820 (2019).

24 Cao, Z., Shu, Y., Qin, H., Su, B. & Peng, X. Quantum Dots with Highly Efficient, Stable, and Multicolor Electrochemiluminescence. *ACS Cent Sci* **6**, 1129-1137 (2020).

25 Zou, R., Lin, Y. & Lu, C. Nitrogen Vacancy Engineering in Graphitic Carbon Nitride for Strong, Stable, and Wavelength Tunable Electrochemiluminescence Emissions. *Anal. Chem.* **93**, 2678-2686 (2021).

26 Wang, F. *et al.* Intrinsic “Vacancy Point Defect” Induced Electrochemiluminescence from Coreless Supertetrahedral Chalcogenide Nanocluster. *J. Am. Chem. Soc.* **138**, 7718-7724 (2016).

27 Chen, S. *et al.* Near Infrared Electrochemiluminescence of Rod-Shape 25-Atom AuAg Nanoclusters That Is Hundreds-Fold Stronger Than That of $Ru(bpy)_3$ Standard. *J. Am. Chem. Soc.* **141**, 9603-9609 (2019).

28 Chen, A. *et al.* Anodic Electrochemiluminescence of Carbon Dots Promoted by Nitrogen Doping and Application to Rapid Cancer Cell Detection. *Anal. Chem.* **92**, 1379-1385 (2019).

29 Carrara, S., Aliprandi, A., Hogan, C. F. & De Cola, L. Aggregation-Induced Electrochemiluminescence of Platinum(II) Complexes. *J. Am. Chem. Soc.* **139**, 14605-14610 (2017).

30 Wang, T., Wang, D., Padelford, J. W., Jiang, J. & Wang, G. Near-Infrared Electrogenerated Chemiluminescence from Aqueous Soluble Lipoic Acid Au Nanoclusters. *J. Am. Chem. Soc.* **138**, 6380-6383 (2016).
31 Wang, F. *et al.* Precise mono-Cu^+ Ion Doping Enhanced Electrogenerated Chemiluminescence from Cd–In–S Supertetrahedral Chalcogenide Nanoclusters for Dopamine Detection. *Nanoscale* **10**, 15932-15937 (2018).
32 Hu, G.-B. *et al.* Highly Stable Ru-complex-grafted 2D Metal-Organic Layer with Superior Electrochemiluminescent Efficiency as A Sensing Platform for Simple and Ultrasensitive Detection of Mucin 1. *Biosens. Bioelectron.* **135**, 95-101 (2019).
33 Li, S., Liu, Y. & Ma, Q. A Novel Polydopamine Electrochemiluminescence Organic Nanoparticle-based Biosensor for Parathyroid Hormone Detection. *Talanta* **202**, 540-545 (2019).
34 Qin, X., Xu, X., Lu, J. & Zhu, Y. Highly Efficient Electrochemiluminescence of Quinoline and Isoquinoline in Aqueous Solution. *Electrochem Commun* **101**, 19-22 (2019).
35 O'Reilly, E. J., Keyes, T. E., Forster, R. J. & Dennany, L. Insights into Electrochemiluminescent Enhancement Through Electrode Surface Modification. *The Analyst* **138**, 677-682 (2013).
36 Chen, A., Zhao, M., Zhuo, Y., Chai, Y. & Yuan, R. Hollow Porous Polymeric Nanospheres of a Self-Enhanced Ruthenium Complex with Improved Electrochemiluminescent Efficiency for Ultrasensitive Aptasensor Construction. *Anal. Chem.* **89**, 9232-9238 (2017).
37 Qin, X. *et al.* A Graphene-like N-annulated Perylene Diimide Dimer Compound for Highly Efficient Electrochemiluminescence. *Electrochim Acta* **450** (2023).
38 Guo, Y.-Z. *et al.* Nitrogen-, Sulfur-, and Fluorine-Codoped Carbon Dots with Low Excitation Potential and High Electrochemiluminescence Efficiency for Sensitive Detection of Matrix Metalloproteinase-2. *Anal. Chem.* **95**, 7021-7029 (2023).
39 Fang, Y. *et al.* Highly Efficient Wavelength-Resolved Electrochemiluminescence of Carbon Nitride Films for Ultrasensitive Multiplex MicroRNA Detection. *Anal. Chem.* **95**, 6620-6628 (2023).
40 Hou, Y. *et al.* Growth of Robust Carbon Nitride Films by Double Crystallization with Exceptionally Boosted Electrochemiluminescence for Visual DNA Detection. *Adv. Opt. Mater.* **11**, 2202737 (2023).
41 Nam, Y.-S. *et al.* Sensitive and Selective Determination of Ion in Aqueous Samples Using Modified Gold Nanoparticle As A Colorimetric Probe. *Talanta* **125**, 153-158 (2014).
42 Li, W. *et al.* Fluorescent Recognition and Selective Detection of Nitrite Ions with Carbon Quantum Dots. *Anal. Bioanal. Chem.* **412**, 993-1002 (2020).
43 Mokhtar, B., Kandiel, T. A., Ahmed, A. Y. & Komy, Z. R. New Application for TiO_2 P25 Photocatalyst: A Case Study of Photoelectrochemical Sensing of Nitrite Ions. *Chemosphere* **268**, 128847 (2021).

44 Muthuchamy, N., Lee, K. P. & Gopalan, A. I. Enhanced Photoelectrochemical Biosensing Performances for Graphene (2D) – Titanium Dioxide Nanowire (1D) Heterojunction Polymer Conductive Nanosponges. *Biosens. Bioelectron.* **89**, 390-399 (2017).

45 Haldorai, Y. *et al.* Direct Electrochemistry of Cytochrome c Immobilized on Titanium Nitride/Multi-Walled Carbon Nanotube Composite for Amperometric Nitrite Biosensor. *Biosens. Bioelectron.* **79**, 543-552 (2016).

46 Vilian, A. T. E. *et al.* Immobilization of Myoglobin on Au Nanoparticle-decorated Carbon Nanotube/Polytyramine Composite as A Mediator-free H_2O_2 and Nitrite Biosensor. *Sci Rep* **5**, 18390 (2015).

47 Singh, L. & Ranjan, N. Highly Selective and Sensitive Detection of Nitrite Ion by an Unusual Nitration of a Fluorescent Benzimidazole. *J. Am. Chem. Soc.* **145**, 2745-2749 (2023).

48 Khachornsakkul, K., Del-Rio-Ruiz, R., Creasey, H., Widmer, G. & Sonkusale, S. R. Gold Nanomaterial-Based Microfluidic Paper Analytical Device for Simultaneous Quantification of Gram-Negative Bacteria and Nitrite Ions in Water Samples. *ACS Sens.* **8**, 4364-4373 (2023).

49 Rajab, N., Ibrahim, H., Hassan, R. Y. A. & Youssef, A. F. A. Selective Determination of Nitrite in Water and Food Samples using Zirconium Oxide (ZrO_2)@MWCNTs Modified Screen Printed Electrode. *RSC Adv.* **13**, 21259-21270 (2023).

50 Xiao, N. & Yu, C. Rapid-Response and Highly Sensitive Noncross-Linking Colorimetric Nitrite Sensor Using 4-Aminothiophenol Modified Gold Nanorods. *Anal. Chem.* **82**, 3659-3663 (2010).

51 Wang, L., Li, B., Zhang, L., Zhang, L. & Zhao, H. Fabrication and Characterization of A Fluorescent Sensor based on Rh 6G-functionlized Silica Nanoparticles for Nitrite Ion Detection. *Sensor Actuat B: Chem* **171-172**, 946-953 (2012).

52 Annalakshmi, M., Kumaravel, S., Chen, S.-M., Balasubramanian, P. & Balamurugan, T. S. T. A Straightforward Ultrasonic-assisted Synthesis of Zinc Sulfide for Supersensitive Detection of Carcinogenic Nitrite Ions in Water Samples. *Sensor Actuat B: Chem* **305** (2020).

53 Nam, J. *et al.* A Colorimetric Hydrogel Biosensor for Rapid Detection of Nitrite Ions. *Sensor Actuat B: Chem* **270**, 112-118 (2018).
